# A Self‐Accelerating Naphthalimide‐Based Probe Coupled with Upconversion Nanoparticles for Ultra‐Accurate Tri‐Mode Visualization of Hydrogen Peroxide

**DOI:** 10.1002/advs.202309182

**Published:** 2024-01-19

**Authors:** Yanan Feng, Da Lei, Baiyi Zu, Jiguang Li, Yajuan Li, Xincun Dou

**Affiliations:** ^1^ Hebei Provincial Key Laboratory of Photoelectric Control on Surface and Interface College of Science Hebei University of Science and Technology Shijiazhuang 050018 China; ^2^ Xinjiang Key Laboratory of Trace Chemical Substances Sensing Xinjiang Technical Institute of Physics and Chemistry Chinese Academy of Sciences Urumqi 830011 China; ^3^ Key Laboratory of Improvised Explosive Chemicals for State Market Regulation Urumqi 830011 China; ^4^ Center of Materials Science and Optoelectronics Engineering University of Chinese Academy of Sciences Beijing 100049 China

**Keywords:** explosive detection, fluorescent probes, hydrogen peroxide, tri‐mode, visualization

## Abstract

The design and development of ultra‐accurate probe is of great significance to chemical sensing in complex practical scenarios. Here, a self‐accelerating naphthalimide‐based probe with fast response and high sensitivity toward hydrogen peroxide (H_2_O_2_) is designed. By coupling with the specially selected upconversion nanoparticles (UCNPs), an ultra‐accurate colorimetric‐fluorescent‐upconversion luminescence (UCL) tri‐mode platform is constructed. Owing to the promoted reaction process, this platform demonstrates rapid response (< 1 s), an ultra‐low detection limit (4.34 nM), and superb anti‐interferent ability even in presence of > 21 types of oxidants, explosives, metallic salts, daily compounds, colorful or fluorescent substances. In addition, the effectiveness of this design is further verified by a sponge‐based sensing chip loaded with the UCNPs/probe in recognizing trace H_2_O_2_ vapor from interferents with the three characteristic colors existing simultaneously. The proposed design of probe and tri‐mode visualization detection platform is expected to open up a brand‐new methodology for ultra‐accurate sensing.

## Graphical abstract

A tri‐mode methodology based on colorimetric, fluorescent and upconversion luminescence has been proposed, which is anticipated to guide future development of ultra‐accurate detection. Representative improvised explosive triacetone triperoxide and its precursor hydrogen peroxide could be identified under extremely complex circumstances by exactly coupling the designed self‐accelerating naphthalimide‐based probe with specific upconversion nanoparticles.

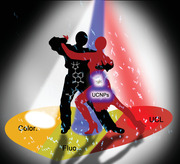

## 1 Introduction

Fluorescent visualization detection technique, which has been employed as a preferable strategy for explosives, illegal narcotics or environmental pollutant detection due to the advantages of high sensitivity, fast response, strong specificity, and visualization of the detection signal.^[^ ^1^, ^2^ ^]^ To date, various fluorescent materials have been explored for high‐performance sensing, such as organic small‐molecule fluorescent probes,^[^ ^3^ ^]^ fluorescent conjugated polymers,^[^ ^4^ ^]^ metal‐organic frameworks (MOFs),^[^ ^5^ ^]^ quantum dots (QDs),^[^ ^6^ ^]^ noble metal nanoparticles,^[^ ^7^ ^]^ and metal nanoclusters.^[^ ^8^ ^]^ Among them, the organic small‐molecule fluorescent probes, which have a series of merits such as rationally designed functional groups, specific recognition sites, adjustable fluorophore structures, as well as changeable emission band and efficiency, have attracted considerable attention.^[^ ^9^ ^]^ It has been demonstrated that simply and effectively improve the detection accuracy of organic small‐molecule fluorescent probes, i.e., boost the detection limit and anti‐interference, is the core goal for trace sensing in complex environments.^[^ ^10^ ^]^ In order to obtain a highly sensitive and selective fluorescent probe, significant attention has been devoted to regulating the interaction forces between target and probe,^[^ ^11^, ^12^ ^]^ i.e., modulating the recognition site of the probe,^[^ ^13^ ^]^ adjusting the electron/energy transfer process,^[^ ^14^, ^15^ ^]^ etc. Especially, as a vital target concerned in explosives detection, human health, food processing, and environmental disinfection, hydrogen peroxide (H_2_O_2_), its probe design has been devoted to a lot of attempts to achieve a more efficient design.^[^ ^16^ ^]^ Especially, based on the different reaction mechanisms such as boric acid oxidation reaction^[^ ^17^ ^]^ and Baeyer‐Villiger oxidation rearrangement reaction,^[^ ^18^ ^]^ and different sensing mechanisms including photo‐induced electron transfer (PET),^[^ ^19^ ^]^ intramolecular charge transfer (ICT),^[^ ^20^ ^]^ and fluorescence resonance energy transfer (FRET).^[^ ^21^ ^]^ Although significant progress has been made to improve the sensitivity, the lack of strong anti‐interference performance in complicate scenarios is still calling for the appearance of a new sensing concept.

Practically, the visualized detection signal from organic small‐molecule fluorescent probes is inevitably disturbed by interferents with fluorescent emissions, such as dust, fluorescent dyes, or plants,^[^ ^22^ ^]^ and even for the background. One efficient strategy to break this bottleneck is to construct a dual‐mode visualization detection platform with two independent signals, such as the colorimetric‐fluorescent dual‐mode detection.^[^ ^23^, ^24^ ^]^ A series of studies proved that fluorophores, such as rhodamine‐based compounds,^[^ ^25^ ^]^ 1,8‐naphthalimide derivatives,^[^ ^26^ ^]^ cyanine derivatives,^[^ ^27^ ^]^ and bodipy‐based derivatives,^[^ ^28^ ^]^ can be used for it. In addition, nanoparticles modified with organic small‐molecule fluorescent probes, such as QDs,^[^ ^29^ ^]^ metal‐based nanoparticles,^[^ ^30^ ^]^ and MOFs,^[^ ^31^ ^]^ have been introduced to construct the dual‐mode nanoprobes.^[^ ^32^ ^]^ However, most of these probes use the colorimetric and downconversion fluorescent signals, which still suffer from the low signal‐to‐background ratio as well as interference by the colorful and fluorescent substances.^[^ ^33^, ^34^ ^]^ In comparison, due to the strong anti‐interference ability to environmental background, upconversion nanoparticles (UCNPs) have emerged as a promising sensing material to fabricate nanoprobes for detecting chemicals based on FRET^[^ ^35^ ^]^ and inner filter effect (IFE).^[^ ^36^ ^]^ Among them, IFE is a phenomenon wherein the photons emitted from the UCNPs are subsequently reabsorbed by the acceptor molecules, leading to the suppression of the upconversion luminescence (UCL) emission. There are two prerequisites to be considered for the construction of an IFE system, first the donor's emission spectrum and the acceptor's absorption spectrum require a large spectral overlap, second the donor should have a sufficiently high quantum yield, and the acceptor should possess broad absorption cross section and large molar extinction coefficient. However, the sensing signal from the UCL could also be influenced by the widely applicated long afterglow materials in traffic signs, safety signs, as well as the overalls of sanitation workers in daily life.^[^ ^37^ ^]^ Therefore, is there any possibility to explore a multi‐mode detection system based on organic small‐molecule probes and UCNPs with both high sensitivity and strong anti‐interference performances, presents a challengeable but attractive direction.

Herein, we report a straightforward strategy to construct an ultra‐accurate tri‐mode visualization platform through colorimetric, fluorescent and UCL visualization analysis based on an elaborated designed organic small‐molecule probe coupled with picked UCNPs through IFE. Specifically, a self‐accelerating N‐n‐hexanoic acid‐4‐boronic acid pinacol ester‐1,8‐naphthimide probe (B‐R‐COOH) with boronate ester as the recognition site for H_2_O_2_ and with the auxiliary carboxylic acid group (−COOH) as the promotion group, was designed and NaYF_4_: Yb, Tm UCNPs were selected to construct the tri‐mode nanoprobe (UCNPs/B‐R‐COOH). Distinct sensing signals, including the gold color under natural light, doderblue fluorescence under 468 nm excitation, and red UCL emission under 980 nm irradiation presented after the addition of H_2_O_2_. The −COOH triggers the self‐acceleration of the detection reaction process and enables an ultra‐low detection limit (4.34 nM) and ultra‐rapid response (< 1 s), as well as the perfect cross discrimination capability in complex scenarios with colored, fluorescent and red long afterglow substances coexisting. We expect the proposed tri‐mode visualization detection concept would pave a brand‐new way for ultra‐accurate sensing platform design.

## 2 Results and Discussion

### 2.1 Design of the Self‐Accelerating Probe for Tri‐Mode Visualization Platform

**Figure 1:**
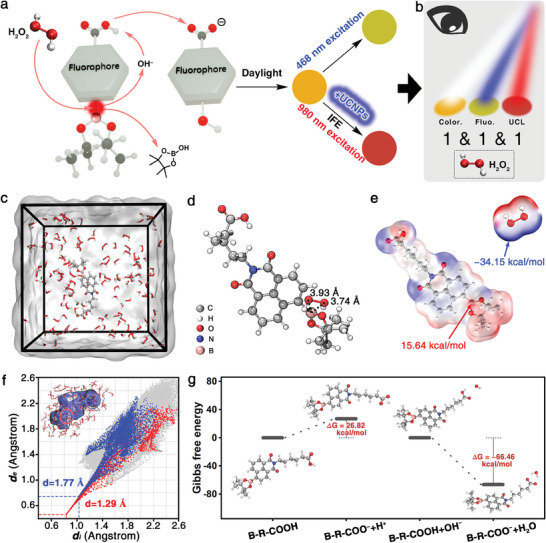
Schematic illustration of a) design strategy for the B‐R‐COOH probe to detect H_2_O_2_ through colorimetric and fluorescent channels, as well as the UCL channel with the aid of UCNPs and b) the visualization judgement criteria of H_2_O_2_ by the logical discriminant method. c) The simulation model constructed via the B‐R‐COOH probe and H_2_O_2_. d) Bond lengths between the B of the B‐R‐COOH probe and O of H_2_O_2_ (black dashed lines). e) The electrostatic potential distribution diagram of the B‐R‐COOH probe and H_2_O_2_. f) The 2D fingerprint plots for the local contact surface of the B‐R‐COOH probe with H_2_O_2_, and the 3D Hirshfeld surfaces model of the H_2_O_2_ molecules surrounding the B‐R‐COOH probe (inset, blue: no attraction, white: weak attraction, red: strong attraction). g) Potential energy profiles for the dissociation of −COOH on the B‐R‐COOH probe as well as the reaction between the −COOH and OH^−^ from the product of the H_2_O_2_‐mediated oxidation of aryl boronates.

In the self‐accelerating B‐R‐COOH, a −COOH locates at the N‐position as the promotion group, a boronate ester acts as the recognition site and the naphthalimide bechaves as the fluorophore (**Figure** **1**a). The detection mechanism lies in the boronate ester group, which can be oxidized and transformed to phenol upon reaction with H_2_O_2_, thus forming an electron donor‐acceptor structure which further turns the ICT transition. Benefiting from the −COOH, the reaction process of the probe toward H_2_O_2_ will be efficiently facilitated via the formation of H_2_O between the hydrogen ion (H^+^) from −COOH and hydroxide anion (OH^−^) from the other product of the H_2_O_2_‐mediated oxidation of aryl boronates. As a result, the detection product with a distinct color under daylight and a strong fluorescence emission under 468 nm excitation could be achieved owing to the ICT transition. Furthermore, by coupling with the design‐orientated UCNPs with the B‐R‐COOH probe, a tri‐mode visualization detection platform could be constructed. Once contacting with H_2_O_2_, it can not only exhibit characteristic color and fluorescence, but also emit characteristic UCL based on IFE. Thus, the tri‐mode visualization detection platform can precisely recognize trace H_2_O_2_ vapor by a logical discriminant method (Figure 1b).

To verify the crucial role of −COOH for detecting H_2_O_2_, the time‐depending interaction between the B‐R‐COOH probe and H_2_O_2_ was evaluated within 20 ns via molecular dynamics (MD) simulations (Figure 1c). It can be observed that the O atom of H_2_O_2_ is closely adjacent to the B atom of boronate group on the B‐R‐COOH probe with bond lengths of 3.74 and 3.93 Å, indicating the formation of a relatively strong interaction between the boronate ester and the H_2_O_2_ molecules (Figure 1d). The electrostatic potential distribution maps indicate the B atom on the B‐R‐COOH probe has the maximum electrostatic potential (15.64 kcal mol^−1^), proving that this electrophile can easily be attacked by the O of H_2_O_2_ owning to the minimum electrostatic potential (−34.15 kcal mol^−1^) (Figure 1e). To quantitatively explore the competitive interaction of the boronate group and other groups on B‐R‐COOH with H_2_O_2_, the Hirshfeld surfaces of H_2_O_2_ surrounding the B‐R‐COOH probe were mapped by electron density (Figure 1f). It is found the obvious red area centers on the boronate group, while a relatively blue area scatters across the surface of the −COOH group, implying that the formation of a strong interaction between the boronate group and H_2_O_2_ and the −COOH group has no interference to the interaction (insert of Figure 1f). In order to precisely illustrate the interactions between the different parts of the probe and H_2_O_2_, the fingerprint plots of the boronate group and −COOH were mapped by combining the distance from the internal (defined as *d_i_*) and external (defined as *d_e_*) nearest atom to the Hirshfeld surfaces. It is obvious the discrete points closely focused on the top of the spike with a total minimum value (d) of 1.29 Å in the boronate group, which forms a sharp contrast with that in the −COOH (1.77 Å), indicating that the interaction between the boronate group and H_2_O_2_ is much stronger than that of the −COOH. Thus, it is obviously verified the strong attraction between the boronate group and H_2_O_2_ can trigger their preferential combination, greatly promoting the H_2_O_2_‐mediated oxidation of aryl boronates and generating the fluorescent product and OH^−^.

To further verify the feasibility of −COOH consuming the OH^−^ through the acid‐base neutralization, the reaction potential energies during the ionization of the B‐R‐COOH and the reaction between the probe and OH^−^ were investigated through thermodynamic and kinetic density functional theory analyses (Figure 1g). It could be observed that the −COOH can easily ionize to produce B‐R‐COO^−^ and H^+^ with a relatively low Gibbs free energy difference (ΔG = 26.82 kcal mol^−1^), and then H^+^ can spontaneously involve in the acid‐base neutralization reaction to OH^−^ with a thermodynamically dominant ΔG as −66.46 kcal mol^−1^. These results indicate that the preferential combination between boronate group and H_2_O_2_ as well as the continued consumption of the OH^−^ through −COOH synergetically drive the reaction forward, proving the superiority of the self‐accelerating probe for ultra‐accurate detection of H_2_O_2_.

### 2.2 The Crucial Role of −COOH on the Detection Performance Toward H_2_O_2_

**Figure 2:**
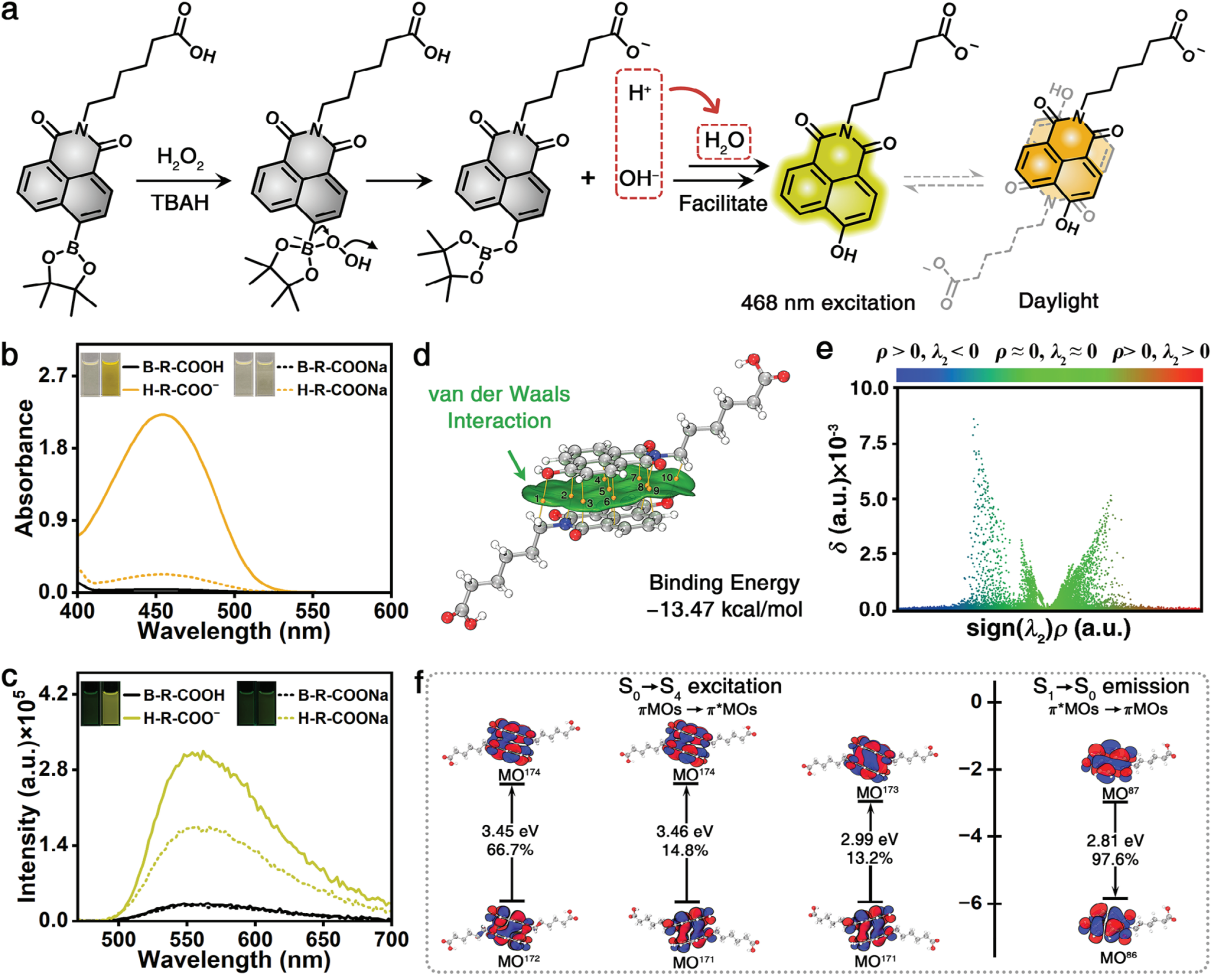
a) Detection mechanism of the designed B‐R‐COOH probe for recognizing H_2_O_2_ via colorimetric (daylight) and fluorescent (468 nm excitation) dual‐mode. b) The UV–vis absorption and c) the fluorescent spectra as well as the corresponding images of the B‐R‐COOH and B‐R‐COONa probes before and after detecting H_2_O_2_. d) The independent gradient model and Atoms‐In‐Molecules topological analysis of the intermolecular interactions of the H‐R‐COO^−^ dimer with optimized configuration. e) The scatter graph of the noncovalent interactions between the H‐R‐COO^−^ dimer. f) Dominant contributions of molecular orbital transitions in the process of interaction: S_0_→S_4_ excitation and S_1_→S_0_ emission.

To systematically assess the role of the −COOH to improve the detection performance toward H_2_O_2_, the detection mechanism of the probe was further investigated. First, the boronate group as a strong electrophile could be attacked by nucleophile hydroperoxide anion originating from the dissociation of H_2_O_2_ with the assistance of tetrabutylammonium hydroxide (TBAH)^[^ ^38^ ^]^ (**Figure** **2**a). Second, the forming of the negatively charged tetrahedral boronate complex proceeds via Bayer‐Villiger oxidation‐like rearrangement and hydrolysis to generate OH^−^.^[^ ^39^ ^]^ Therefore, −COOH could efficiently facilitate the sensing reaction via reacting with OH^−^ to form H_2_O, which can be defined as a self‐accelerating process. Simultaneously, the aryl boronate group could be transformed to phenol, which is a strong electron donor, thus effectively enhances the ICT absorption and emission of the product N‐n‐hexanoic acid‐4‐hydroxy‐1,8‐naphthimide anion (H‐R‐COO^−^), corresponding to an obvious color and fluorescence emission.

To clearly demonstrate role of −COOH, the B‐R‐COOH and sodium N‐n‐hexanoic acid‐4‐boronic acid pinacol ester‐1,8‐naphthimide (B‐R‐COONa) probes as well as the H‐R‐COO^−^ product were synthesized and characterized (Scheme S1, Figures S1–S7, Supporting Information). Meanwhile, the comparison of the sensing performance between the above two probes toward H_2_O_2_ was assessed. From the view of colorimetric channel (Figure 2b), it is clear that both probes show almost no absorption from 400 to 600 nm with a similar pale yellow color. While the characteristic absorption peak at ≈454 nm reveals an obvious enhancement to different degrees upon the addition of H_2_O_2_ (600 mM) due to the formation of H‐R‐COO^−^ and sodium N‐n‐hexanoic acid‐4‐hydroxy‐1,8‐naphthimide (H‐R‐COONa), respectively. It is noteworthy that the absorption intensity of H‐R‐COO^−^ is ≈11.5 times of that of H‐R‐COONa, and the corresponding colors are gold and light yellow, indicating that the −COOH is more effective in promoting the detection performance. In the fluorescent channel (Figure 2c), it is obvious that there is almost no difference in the peak position (with the emission band at 558 nm in a green color) and intensity under 468 nm excitation between them. While the fluorescent emission located at 558 nm displays a distinct enhancement after adding H_2_O_2_ (600 mM), and the corresponding fluorescent intensity of H‐R‐COO^−^ in doderblue is ≈1.9 times of that of H‐R‐COONa in green. Notably, due to the −COOH group‐facilitated formation of H‐R‐COO^−^, the B‐R‐COOH probe toward H_2_O_2_ is obviously superior than that of the B‐R‐COONa probe in both response modes. Therefore, it is sufficiently proved from theory and experiment that the unique B‐R‐COOH probe with −COOH endows the excellent colorimetric‐fluorescent dual‐mode sensing performance toward H_2_O_2_.

To explain the difference of the enhancement ratio between absorption and fluorescence before and after detection, the origins of the absorption and fluorescence of the H‐R‐COO^−^ product were further investigated. From the excited state calculations of the H‐R‐COO^−^ monomer, no obvious absorption peak can be observed in the visible light region because there is no new chromophore group (Figure S8a, Supporting Information). However, a strong fluorescent emission peak of the H‐R‐COO^−^ monomer can be observed at 444 nm with a large oscillator strength (ƒ = 0.2511) (Figure S8b, Supporting Information), which is blue‐shifted ≈100 nm compared with the experimental fluorescence emission peak, the corresponding error is within a reasonable range of the theoretical calculation.^[^ ^40^ ^]^ Significantly, the naphthalimide group of the H‐R‐COO^−^ monomer possesses a relatively good conjugated planar structure, which may form dimer through π‐π stacking interactions with each other. It could be found that a large and continuous green isosurface appears between the naphthalimide groups of the H‐R‐COO^−^ dimer based on the independent gradient model visualized analysis (Figure 2d). And the corresponding binding energy (−13.47 kcal mol^−1^) is far below that of B‐R‐COOH dimer (−5.6 kcal mol^−1^) (Table S1, Supporting Information), indicating the existence of the relatively strong van der Waals interactions in the H‐R‐COO^−^ dimer. Furthermore, based on the Atoms‐In‐Molecules topological analysis, the intermolecular attraction forces between the two monomers were quantitatively demonstrated as the bond critical points (BCPs). It should be noted that the total electron energy density per electron values H(r)/ρ(r) on the BCP can be used to assess the interaction strength, and the H(r)/ρ(r) values inversely proportional to strength of the interaction.^[^ ^41^ ^]^ Ten independent BCPs can be found with the H(r)/ρ(r) within the range of 0.07–0.21 (Figure S9, Supporting Information), representing the strong interaction endowed by the face‐to‐face aromatic π–π stacking of the naphthalimide rings on the H‐R‐COO^−^ products. Furthermore, from the simulation of the weak interaction between the H‐R‐COO^−^ dimer (Figure 2e), it can be seen that the green discrete points mainly distribute around the left of the central sign(*λ*_2_)*ρ* in the scatter plot, demonstrating that the intermolecular force is mainly contributed by the van der Waals force from π‐π stacking. All these π‐π stacking interactions as a whole can prompt the H‐R‐COO^−^ monomer to form the relatively stable H‐R‐COO^−^ dimer. Based on the excited state calculations of the H‐R‐COO^−^ dimer, it can be found that a strong UV–vis absorption appears ranging from 380 to 450 nm, which is close to the experimental data (400–525 nm), while there is almost no fluorescence with extremely low ƒ = 0.0005 (Figure S10, Supporting Information).

To further investigate the source of absorption and fluorescence of the H‐R‐COO^−^ product, the analysis of the excited states molecular orbital (MO) was calculated (Figure 2f). It is observed that the strong UV–vis absorptions derived from the H‐R‐COO^−^ dimer is contributed by the S_0_→S_4_ transition, and it is comprised of at least three pairs of MOs including MO^172^→MO^174^ (π→π* 66.7%), MO^171^→MO^174^ (π→π* 14.8%) and MO^171^→MO^173^ (π→π* 13.2%) with the energy gaps of 3.45, 3.46, and 2.99 eV, respectively. Meanwhile, the fluorescent emission derives from the H‐R‐COO^−^ monomer is ascribed to the S_1_→S_0_ transition from MO^87^→MO^86^ (π*→π 97.6%) with an energy gap of 2.81 eV. All the above results prove that the absorption and fluorescent emission after detection derive from the H‐R‐COO^−^ dimer and monomer, respectively.

To further optimize the sensing performance of the B‐R‐COOH probe, ethanol was chosen as the solvent (Figure S11, Supporting Information), the concentration ratio of TBAH to B‐R‐COOH was selected as 1:1 (Figure S12, Supporting Information), and the concentration of B‐R‐COOH was set as 1 mM (Figure S13, Supporting Information). It can be observed that there is a remarkable and continuous enhancement of the absorption peak appearing at 454 nm by increasing the H_2_O_2_ concentration (Figure S14a, Supporting Information). Meanwhile, the fluorescence intensity gradually increases from 0 to 250 µM, and then decreases from 250 to 600 µM owing to the aggregation‐caused quenching (ACQ) (Figure S14c, Supporting Information). Therefore, the difference of the enhancement ratio between absorption and fluorescence after detection can be ascribed as the obvious ACQ effect. The limits of detections (LODs) of the B‐R‐COOH probe calculated by 3σ/k are 236.84 nM in the range of 0–350 µM in the colorimetric mode and as low as 3.36 nM within the range of 0–80 µM in the fluorescent mode, respectively (Figure S14b,d, Supporting Information). It should be noted that the LOD of the fluorescent mode is much superior to those of the previously reported fluorescence involved methods (Table S2, Supporting Information), demonstrating the great effectiveness of the self‐accelerating −COOH group design.

### 2.3 Characterization of the UCNPs Coupled B‐R‐COOH Nanoprobe

**Figure 3:**
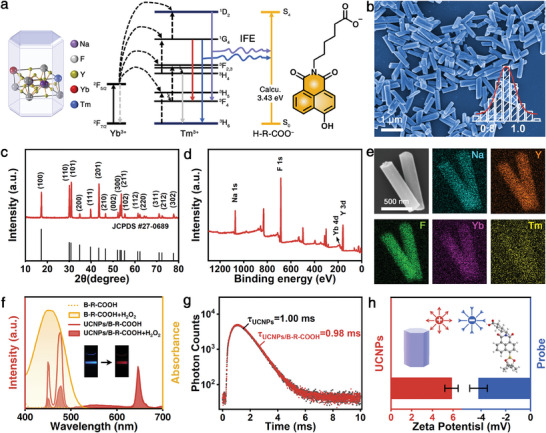
a) Schematic diagrams of energy levels for upconversion processes of UCNPs and the proposed IFE processes upon 980 nm excitation (energy level for H‐R‐COO^−^ taken from theoretical calculating value). b) SEM image and size distribution (inset) of UCNPs. c) XRD pattern of UCNPs. d) XPS spectra of UCNPs. e) Energy‐dispersive X‐ray spectroscopy (EDS) mappings of Na, Y, F, Yb and Tm elements. f) UV–vis absorption spectra of B‐R‐COOH probe and UCL spectra of UCNPs/B‐R‐COOH nanoprobe before and after adding H_2_O_2_, as well as the corresponding UCL photograph under 980 nm excitation (inset). g) Luminescence lifetime of UCNPs (black curve) and UCNPs/B‐R‐COOH nanoprobe (red curve) after adding H_2_O_2_. h) Zeta potential of UCNPs and B‐R‐COOH probe.

The oleic acid (OA)‐coated NaYF_4_: Yb, Tm UCNPs (briefly termed as UCNPs), which can exactly match with the B‐R‐COOH probe in energy through IFE, were introduced to form the UCNPs/B‐R‐COOH nanoprobe. From the energy level diagrams analysis, it can be observed that the emission energy gaps of ^1^D_2_→^3^F_4_ and ^1^G_4_→^3^H_6_ are very close to the energy gap of the S_0_→S_4_ excitation of the H‐R‐COO^−^ dimer. This matching would facilitate an efficient energy transfer from Tm^3+^ to H‐R‐COO^−^ dimer, thus induce a significant UCL color change from blue to red via IFE mechanism (**Figure** **3**a).

Specifically, the UCNPs were obtained through a modified solvothermal method using OA as the surfactant.^[^ ^42^ ^]^ Scanning electron microscopy (SEM) reveals the formation of highly monodispersed and uniform hexagonal prism nanorods with an average length of 0.92 µm (Figure 3b). High‐resolution transmission electron microscopy (HRTEM) image exhibits that the UCNPs are in good crystalline, and the lattice fringes with a *d*‐spacing of 0.513 nm corresponds well with the (100) plane of the hexagonal NaYF_4_ structure (Figure S15, Supporting Information). The main X‐ray diffraction (XRD) peaks are sharp and could be ascribed to the standard hexagonal phase of NaYF_4_ (JCPDS #27‐0689), demonstrating that Yb and Tm have almost no influence on the hexagonal phase and the crystallinity (Figure 3c). In addition, the corresponding X‐ray photoelectron spectroscopy (XPS) verifies the presence of Na, F, Yb and Y (Figure 3d), and the peaks centered at 186.2 and 200.5 eV belong to the Yb 4d_5/2_ and Yb 4d_3/2_, further demonstrating the successful doping of Yb into the NaYF_4_ lattice (Figure S16, Supporting Information). It is also found that Tm could not be observed in the XPS result, which could be attributed to the extremely small ratio in the precursor (0.2%) resulting limited incorporation into the UCNP lattice. Furthermore, Na, Y, F, Yb and Tm uniformly distribute in the observed area by large‐scale elemental mapping analysis, indicating the controllable synthesis of the UCNPs (Figure 3e).

Fourier transform infrared (FT‐IR) spectra provide the evidence of successful fabrication of the oleic acid‐modified UCNPs by the appearance of the characteristic stretching vibrations of −CH_2_− (2925 and 2854 cm^−1^) and −COO− (1562 and 1465 cm^−1^) of OA (Figure S17, Supporting Information). There is almost no absorption for B‐R‐COOH probe in the visible range (Figure 3f), while it exhibits a new and strong absorption band (400 to 525 nm) centered at 454 nm after the addition of H_2_O_2_, which exactly overlaps with the UCL emission bands of UCNPs from 440 to 495 nm. This phenomenon leads to the quenching of the blue fluorescence of UCNPs by the energy transfer from UCNPs to the H‐R‐COO^−^ product. The UCL at 646 nm is not affected and thus causes the UCL color to change from blue to red observed with naked eye. Furthermore, from the luminescence lifetime measurement, it can be seen that the luminescence lifetime of UCNPs without and with the addition of B‐R‐COOH after adding H_2_O_2_ is calculated to be 1.00 and 0.98 ms, respectively, proving the occurrence of the IFE process (Figure 3g). To clearly understand the state of UCNPs and B‐R‐COOH probe in solution, zeta potential analysis was performed, and the results indicate that the surface of UCNPs is positively charged with a zeta potential of 5.85 mV, while B‐R‐COOH probe is −4.20 mV (Figure 3h). In addition, the −C = O (−COOH group) stretching vibration of the B‐R‐COOH probe shifted from 1710 to 1716 cm^−1^ owing to the electrostatic interaction between the UCNPs and B‐R‐COOH probe (Figure S18, Supporting Information). Furthermore, the electrostatic binding energy between the UCNPs and B‐R‐COOH probe is as large as −2.77 eV according to a systematical theoretical analysis (Figure S19 and Table S3, Supporting Information). These results indicate that the UCNPs and B‐R‐COOH probe electrostatically interact with each other and form a relatively uniform dispersion, which is highly desirable for liquid phase detection.

### 2.4 Colorimetric‐Fluorescent‐UCL Tri‐Mode Sensing of H_2_O_2_ Solution

**Figure 4:**
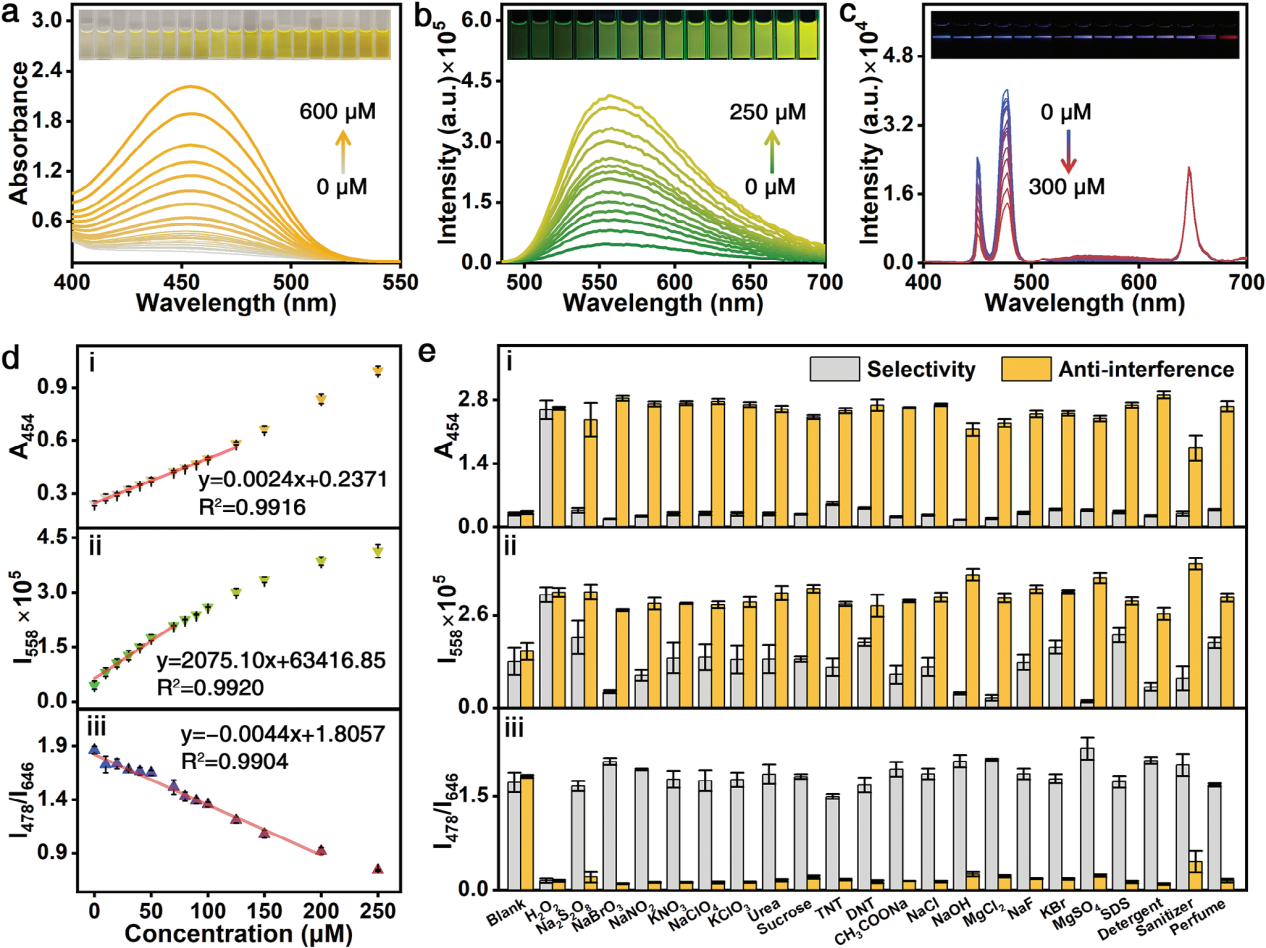
The optical spectra and the corresponding images (inset) from UCNPs/B‐R‐COOH nanoprobe toward H_2_O_2_ under a) colorimetric, b) fluorescent, and c) UCL modes. d) i) absorption intensity (A_454_), ii) fluorescence intensity (I_558_), and iii) UCL intensity ratio (I_478_/I_646_) as a function of H_2_O_2_ concentration. e) The comparison of i) absorption intensity (A_454_), ii) fluorescence intensity (I_558_), iii) UCL intensity ratio (I_478_/I_646_) obtained for UCNPs/B‐R‐COOH nanoprobe in response to various analytes (gray column) and a mixture of H_2_O_2_ and various analytes (orange column). Note: the above spectra and optical images were obtained under natural light (colorimetric mode), the excitation of 468 nm (fluorescent mode) and 980 nm (UCL mode), respectively. The standard deviations were obtained from three repeated measurements.

To improve the detection performance of the UCNPs/B‐R‐COOH nanoprobe for H_2_O_2_, the optimal concentration of UCNPs was determined as 0.5 mg mL^−1^ (Figure S20, Supporting Information). In the colorimetric channel, when the concentration of H_2_O_2_ varies from 0 to 600 µM, the absorption spectra show a gradual enhancement of the peak (400 to 525 nm) centered at 454 nm with a gentle color change from pale yellow to gold (**Figure** **4**a). In the fluorescent channel, with the increasing of the concentration of H_2_O_2_ (0–250 µM), the fluorescence peak gradually increases along with the change of the emission color from green to doderblue (Figure 4b). The UCL intensities of the nanoprobe at 450 and 478 nm gradually decrease with increasing H_2_O_2_ concentration from 0 to 300 µM while remain almost constant at 646 nm (Figure 4c), displaying ratiometric luminescence emission characteristics varying from blue, purple to red. It's worth noting that there appears a new weak emission from 500 to 600 nm after the addition of H_2_O_2_, which comes from the fluorescence emission of the H‐R‐COO^−^ product excited by the 450 and 478 nm emissions of the UCNPs.

Furthermore, the correlation between the absorption/fluorescence/UCL intensity and the H_2_O_2_ concentration were systematically analyzed and the corresponding LODs of the three channels were calculated by 3σ/k. Specifically, in the colorimetric channel (Figure 4di), the absorption intensity exhibits a good linear behavior with the increase of the H_2_O_2_ concentration from 0 to 125 µM. The LOD in the colorimetric channel is calculated as 375.00 nM (k = 0.0024, σ = 0.0003). Meanwhile, in the fluorescent channel (Figure 4dii), the fitted curve shows an excellent linearity in the range of 0–70 µM, and the LOD could reach as low as 4.34 nM (k = 2075.10, σ = 3), which is similar to that of the B‐R‐COOH probe and much superior than other probes reported previously (Table S2, Supporting Information). In the UCL channel (Figure 4diii), it is found that the ratio of the UCL intensity (I_478_/I_646_) decreases linearly with the increasing of the concentration of H_2_O_2_ from 0 to 200 µM and the LOD is determined as 94.36 nM according to 3σ/(k×$\bar{F}$_646_) (σ = 3, k = 0.0044, $\bar{F}$ is the average luminescence intensity of the nanoprobe at 646 nm with a value of 21 676.45).

To systematically evaluate the specificity and anti‐interference ability of the UCNPs/B‐R‐COOH nanoprobe, more than 21 substances consisting of oxidants, explosives, water‐soluble metallic salts, and daily compounds with 10 times higher concentration were selected as the interferents. Under natural light, the UCNPs/B‐R‐COOH nanoprobe solution shows a remarkable absorption toward H_2_O_2_ with significant intensity enhancement, while the other 21 interferents only lead to little change in the absorption intensity, demonstrating the superior selectivity in the colorimetric channel (Figure 4e i; Figure S21a, Supporting Information). Furthermore, all the interferents have no apparent influence on the final characteristic absorption toward H_2_O_2_, with the absorption intensity maintained at > 82.3%. Even for sanitizer (absorption intensity of 66.6%), it still has no effect on the judgement of the existence of H_2_O_2_ if mixing with H_2_O_2_ (Figure S21b, Supporting Information), implying the superior specificity and the anti‐interference ability of the nanoprobe in the colorimetric channel. Under 468 nm illumination, compared with the sharp enhancement of the fluorescent emission when H_2_O_2_ was added, there were no obvious changes in the fluorescence spectra at 558 nm in the presence of 21 interferents except for Na_2_S_2_O_8_ and sodium dodecyl sulfate (SDS) (Figure 4e ii; Figure S22, Supporting Information). Consequently, Na_2_S_2_O_8_ and SDS unable to be completely distinguished from H_2_O_2_ as the maximum response in value is up to 39.5% of that of H_2_O_2_, however, both of them can be identified based on the color changes in the colorimetric mode. Furthermore, it is observed that the fluorescence‐enhanced phenomenon toward H_2_O_2_ is achieved without an obvious influence with interferents, and the intensities after adding the mixture of interferents and H_2_O_2_ are always maintained at more than 81.6% of that with only H_2_O_2_. Under 980 nm irradiation, the nanoprobe has a superior selectivity response toward H_2_O_2_ (Figure 4e iii). Meanwhile, the UCL images and spectra show no apparent changes in the presence of H_2_O_2_ with 21 interferents co‐existing respectively (Figure S23, Supporting Information), which can be ascribed to the unique upconversion excitation lead nearly zero background. Compared with other previously published probes, UCNPs/B‐R‐COOH nanoprobe has outstanding anti‐interference capability in the visualization detection of H_2_O_2_ (Table S2, Supporting Information). In addition, the entire sensing process can be visualized within 1 s in the three channels by the difference processing between the sensing images and the initial images (Figure S24, Supporting Information). Thus, the UCNPs/B‐R‐COOH nanoprobe presents promising sensitivity, selectivity, anti‐interference capability, and fast response toward H_2_O_2_, making it well applicable for on‐site detection toward H_2_O_2_.

### 2.5 Sensing Chip‐Enabled Tri‐Mode Detection toward H_2_O_2_ Vapor

**Figure 5:**
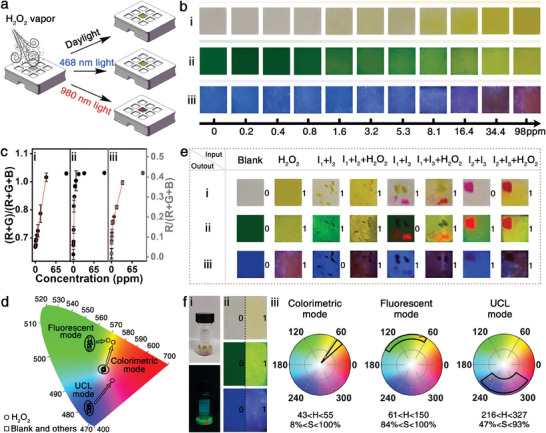
a) Schematic illumination of the UCNPs/B‐R‐COOH nanoprobe loaded sponge‐based sensing chip for detection of H_2_O_2_ vapor. b) The i) colorimetric, ii) fluorescent and iii) UCL optical images after detecting H_2_O_2_ vapor with different concentrations. c) The corresponding plotting curves of i) ii) (R+G)/(R+G+B) value and iii) R/(R+G+B) value extracted from the tri‐mode sensing chip versus the H_2_O_2_ concentration. d) The evaluation of selectivity of the tri‐mode sensing chip based on the CIE 1931 chromaticity diagram. e) The evaluation of anti‐interference ability of the tri‐mode sensing chip to identify H_2_O_2_ from the mixture of H_2_O_2_ and interferents, and the corresponding true table (i: colorimetric mode; ii: fluorescent mode; iii: UCL mode): I_1_: gold pigment, I_2_: doderblue fluorescent powder, I_3_: red long afterglow material. f) Using the tri‐mode sensing chip for i) practically detecting TATP, ii) the images obtained before and after testing under natural light (colorimetric mode), 468 nm (fluorescent mode) and 980 nm (UCL mode) excitation, respectively, and iii) the threshold space of the hue (H) and saturation (S) for three channels extracted from (b) and the corresponding HS plot of TATP vapor.

In consideration of monitoring H_2_O_2_ vapor is vital and significant to screen explosive terrorist activity, a sponge‐based sensing chip with a tri‐mode optical response was constructed by adopting a melamine sponge with no fluorescence emission as the support to immobilize the UCNPs/B‐R‐COOH nanoprobe solution (**Figure** **5**a). This sensing chip design derives from two aspects, the first one is to ensure the efficient anchoring of the UCNPs/B‐R‐COOH nanoprobe through the 3D porous network structure of the sponge, and the other one is to concentrate the color signals on the surface and improve the sensitivity in comparison to the free‐diffusion solution system. It can be observed from the morphology and the corresponding EDS mappings (Figure S25, Supporting Information) that the nanoprobe is uniformly distributed in the sponge. When the sensing chip is exposed to H_2_O_2_ vapor, it is obvious that the color changes from pale yellow to gold, the fluorescent emission changes from green to doderblue, and the UCL emission varies from blue to purple and then to red. Specifically, with the increase of the concentration of H_2_O_2_ vapor from 0 to 98 ppm, the colorimetric, fluorescent, and UCL channels all achieve the most apparent color at 34.4 ppm and basically remain unchanged with further increasing the H_2_O_2_ concentration (Figure 5b). Furthermore, by the numerical analysis of the tri‐mode visualization signals based on the RGB value extraction, the saturation trend is distinctly presented. A great linear relationship in the range from 0 to 34.4 ppm is achieved in the colorimetric mode, and then the value remains at a saturated status after 34.4 ppm (Figure 5ci). While a sharp increase from 0 to 3.2 ppm with a linear relationship could be observed in the fluorescent mode, and the value reaches a plateau after 16.4 ppm (Figure 5cii). In the UCL mode, a sharp rise from 0 to 3.2 ppm with a linear behavior is followed by a relatively gentle increase from 3.2 ppm to 34.4 ppm (Figure 5ciii). It is worth noting that the tri‐mode sensing chip possesses the quantitative sensing capability by the colorimetric mode with a wider linear relationship from 0 to 34.4 ppm levels, and the synergistic judge of the fluorescent mode and the UCL mode can calibrate the value from the colorimetric signal.

To assess the interference of common volatile substances, organic solvents, and daily products on the detection performance in real environment, the selectivity of the tri‐mode sensing chip was investigated by discriminating H_2_O_2_ vapor from 15 types of interferents in the form of vapor (Figure S26, Supporting Information). Upon a quick observation, only the target H_2_O_2_ vapor can lead to the characteristic changes from pale yellow to gold in color, from green to doderblue in fluorescence, and from blue to red in UCL. Moreover, it can be found that the exact positions of the spectral coordinates of these interferents overlapping with each other or forming a separate cluster, are all far away from those of the H_2_O_2_ vapor in the three channels by the CIE 1931 chromaticity diagram analysis (Figure 5d).

Considering colorful and fluorescent matters will inevitably coexist in the actual detection scenes, such as gold pigment, doderblue fluorescent powder and red long afterglow materials, which are similar to the three characteristic signal colors toward H_2_O_2_ vapor, the mixture substances were selected as the interferents (Figure 5e; Figure S27, Supporting Information). The detection result of the tri‐mode sensing chip can be clearly demonstrated with the help of a simple and useful logic design. Specifically, one input represents an analyte, and the specific color change (output) is defined as logical “‘1′”, while others are defined as “‘0′”. Thus, the output code (1, 1, 1) could serve as an indication of having the ingredient of H_2_O_2_, while all the other output codes could be deemed to have no H_2_O_2_ constituent. For the single gold pigment, doderblue fluorescent powder or red long afterglow material mixing with H_2_O_2_, it exhibits the characteristic signal (1) upon daylight, 468 nm light and 980 nm light irradiation, respectively, which is difficult to distinguish in the corresponding detection channel (Figure S27, Supporting Information). Nevertheless, the true signal can be easily discriminated by the signals from two other detection channels, indicating that single interferent has no influence on the detection of H_2_O_2_ by the output code (1,1,1). Even two interferents and H_2_O_2_ exist together, H_2_O_2_ still can be recognized by the signal of the third channel, clearly demonstrating the superior anti‐interference capability of the sensing chip from the mutual authentication.

It is well known that H_2_O_2_ is usually used as a precursor for synthesis of triacetone triperoxide (TATP), an organic peroxide‐based homemade explosive, which also can intrinsically decompose into trace H_2_O_2_ at room temperature.^[^ ^43^ ^]^ To verify the actual detection performance of the tri‐mode sensing chip, a highly complicated practical scenario with the mixture of TATP, gold pigment, doderblue fluorescent powder, red long afterglow material, salt (NaCl), cumin powder, chilli powder, coffee powder, milk‐tea powder, amoxicillin powder, soil and sand was simulated (Figure 5f). Once contacting with the mixture vapor of the above substances, the sensing chip exhibits the characteristic gold color (1), doderblue fluorescence (1) and red UCL (1) in the three channels with negligible influence from the interferents. Furthermore, a characteristic hue (H) and saturation (S) database about the tri‐mode detection signals of H_2_O_2_ was built based on the tri‐mode optical signals after detecting H_2_O_2_ vapor with a series of concentrations. It is clear that the values of H and S of this unknown analyte are exactly located in the range of the characteristic HS database, implying the sensing chip is highly reliable for ultra‐accurate discrimination of trace H_2_O_2_ in real screening scenarios. Benefitting from the fantastic detection sensitivity, ultra‐rapid response, and excellent anti‐interference capability of the tri‐mode sensing chip toward H_2_O_2_ vapor, this sensing chip can be easily incorporated into a tri‐mode portable detector equipped with a white light‐468 nm‐980 nm triple conversion light source for onsite detection. We envisage the designed tri‐mode portable detector is highly promising for searching hidden TATP explosives or H_2_O_2_ based on our previous work on improvised explosives detection (Figure S28, Supporting Information).^[^ ^44^ ^]^

## 3 Conclusion

In conclusion, we have demonstrated the precise design of the self‐accelerating group −COOH is of great significance to boost the detection performance of the B‐R‐COOH probe toward H_2_O_2_ with desirable colorimetric and fluorescence turn‐on signals. By coupling it with the UCNPs, a tri‐mode visualization detection UCNPs/B‐R‐COOH nanoprobe was developed with an ultra‐low detection limit (4.34 nM), rapid response (< 1 s), and excellent anti‐interferent capability. The constructed sensing chip has proven to be reliable for ultra‐accurate detection of TATP in the presence of various mixtures of gold pigment, doderblue fluorescent powder, red long afterglow materials, and even under extremely complex circumstances. We believe that the proposed strategy is a promising candidate for ultra‐accurate and on‐site detection of trace hazardous substances and tri‐mode visualization detection platform conceptualization.

## 4 Experimental Section

### Materials

Unless otherwise noted, all reagents were purchased from commercial sources and used without further purification. 6‐aminocaproic acid was purchased from J&K Scientific (Beijing, China), 4‐bromo‐1,8‐naphthalic anhydride was purchased from Adamas (Shanghai, China), hexylamine and [1,1′‐bis(diphenylphosphino)ferrocene]dichloropalladium(II) (Pd(dppf)Cl_2_) were purchased from Tianjin Heowns Biochemical Technology Co., Ltd. (Tianjin, China), bis(pinacolato)diboron was purchased from Tokyo Chemical Industry (Shanghai) Co., Ltd. (Shanghai, China), 1,4‐dioxane, tetrabutylammonium hydroxide (TBAH, 40% in water), oleic acid (OA, 90%), yttrium(III) chloride hexahydrate (YCl_3_·6H_2_O, 99.9%), thulium(III) chloride hexahydrate (TmCl_3_·6H_2_O, 99.9%), ytterbium(III) chloride hexahydrate (YbCl_3_·6H_2_O, 99.9%) and cyclohexane were purchased from Shanghai Aladdin Biochemical Technology Co., Ltd. (Shanghai, China), and the others inorganic salts and organic solvents were purchased from Sinopharm Chemical Reagent Ltd. (Shanghai, China).

### Characterizations

^1^H NMR and ^13^C NMR spectra were obtained on a Varian 400‐NMR spectrometer with CDCl_3_ or DMSO‐*d_6_* as deuterated solvent. FT‐IR spectra were measured with a PerkinElmer Frontier FT‐IR spectrometer. Mass spectra were obtained on a Q Exactive‐type four stage rod‐Orbitrap high‐resolution mass spectrometer (HRMS, UHPLC‐Q‐Orbitrap‐HRMS, Thermo Fisher Scientific). Fluorescence spectra, UCL spectra and luminescence lifetime of UCNPs were determined by the Edinburgh FLS1000 fluorescence spectrophotometer. UV‐vis absorption spectra were determined with the Hitachi UV‐3900 ultraviolet‐visible spectrophotometer. TEM (JEOL JEM‐F200, Japan) and FE‐SEM (JEOL JSM‐7610F Plus) equipped with an energy‐dispersive X‐ray spectrometer were used for morphology and composition characterization of the UCNPs. The XRD data were measured on the Bruker D8 Advance, with Cu‐K_α_ radiation operating at 40 kV and 40 mA. XPS spectra were measured on the Thermo Scientific Escalab 250Xi, equipped with an Al K_α_ (1486.6 eV) X‐ray source. Zeta potential was measured on the Zeta potential analyzer (Malvern Zetasizer Nano ZS90). The optical photographs were taken on a Realme GT Master Explore smartphone, under natural light, 468 nm (FC‐980‐30 W, CNIlaser) and 980 nm (FC‐980‐30 W, CNIlaser) excitation, respectively.

### Calculation Section

All calculations based on the quantum chemistry were performed using the Gaussian 09 Revision A.02.^[^ ^45^ ^]^ The calculations based on the MD simulation were performed using GROMACS 2022.3 source code.^[^ ^46^ ^]^ The Multiwfn 3.8 (dev) software^[^ ^47^ ^]^ was used for further wavefunction analysis, including the IGMH analysis.^[^ ^48^ ^]^ The Multiwfn 3.8 (dev) and the VMD software^[^ ^49^ ^]^ were used for rendering all the graphs. More details were provided in the Supporting Information.

## Conflict of Interest

The authors declare no conflict of interest.

## Supporting information

Supporting Information

## Data Availability

The data that support the findings of this study are available from the corresponding author upon reasonable request.
