# Supplementary material for: A Self‐Accelerating Naphthalimide‐Based Probe Coupled with Upconversion Nanoparticles for Ultra‐Accurate Tri‐Mode Visualization of Hydrogen Peroxide
Source: Adv Sci (Weinh). 2024 Jan 19;11(13):2309182. doi: 10.1002/advs.202309182 (PMC10987149; doi:10.1002/advs.202309182)
Supplement: Supplementary file 1 — Supporting Information [file ADVS-11-2309182-s001.pdf]

ADVANCED SCIENCE
Open Access

Supporting Information

for *Adv. Sci.*, DOI 10.1002/advs.202309182

A Self-Accelerating Naphthalimide-Based Probe Coupled with Upconversion Nanoparticles for Ultra-Accurate Tri-Mode Visualization of Hydrogen Peroxide

Yanan Feng, Da Lei, Baiyi Zu, Jiguang Li, Yajuan Li* and Xincun Dou**

Supporting Information

A Self-accelerating Naphthalimide-based Probe Coupled with Upconversion Nanoparticles for Ultra-accurate Tri-mode Visualization of Hydrogen Peroxide

Yanan Feng, Da Lei, Baiyi Zu, Jiguang Li, Yajuan Li,* and Xincun Dou**

1. Synthesis process of probe

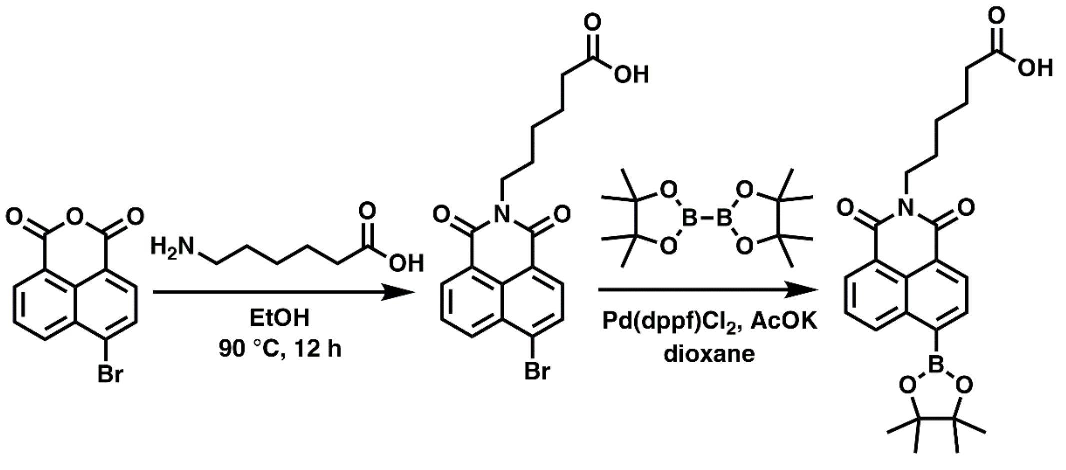

Scheme S1. Synthetic procedures of the B-R-COOH probe.

1.1. Synthesis of N-n-hexanoic acid-4-Bromo-1,8-naphthimide (1)

4-Bromo-1,8-naphthalic anhydride (554.5 mg, 2 mmol), 6-Aminocaproic acid (328.6 mg, 2.5 mmol) and ethanol (30 mL) were mixed in a 100 mL single-necked flask, and the mixture solution was refluxed at 90 °C for 12 h under stirring. After cooling to room temperature, precipitation of the product was carried out from the solution by adding ice water, then the product was filtered and rinsed with deionized water for three times. The resulting solid product was purified on a silica-gel column using dichloromethane: methanol (30:1 by volume, R_f = 0.2) as eluent and dried in vacuum. The desired intermediate product N-n-hexanoic acid-4-Bromo-1,8-naphthimide (**1**) was obtained with 60% yield. ^{1}H NMR (400 MHz, $CDCl_3$) δ (ppm): 8.66 (d, J = 7.3 Hz, 1H), 8.57 (d, J = 8.5 Hz, 1H), 8.41 (d, J = 7.9 Hz, 1H), 8.04 (d, J = 7.9 Hz, 1H), 7.88 – 7.82 (m, 1H), 4.20 – 4.14 (m, 1H), 2.41 – 2.34 (m, 2H), 1.74 (dq, J = 23.1, 7.6 Hz, 4H), 1.53 – 1.45 (m, 2H). ^{13}C NMR (101 MHz, $CDCl_3$) δ (ppm): 178.39, 163.62, 133.28, 132.07, 131.19, 130.65, 130.27, 129.02, 128.09, 123.10, 122.23, 40.26, 33.65, 27.66, 26.49, 24.35. HRMS: $[M]^+$ Calcd. for 390.23; Found 391.04.

1.2. Synthesis of N-n-hexanoic acid-4-boronic acid pinacol ester-1,8-naphthimide (B-R-COOH) and sodium N-n-hexanoic acid-4-boronic acid pinacol ester-1,8-naphthimide (B-

R-COONa)

A mixture of the intermediate product **1** (389.7 mg, 1 mmol), bis(pinacolato)diboron (337.5 mg, 1.5 mmol), Pd(dppf)Cl_2 (26.7 mg, 0.06 mmol), potassium acetate (414.2 mg, 3 mmol) and 1,4-dioxane (15 mL) were refluxed at 90 °C with stirring for 12 h under nitrogen protection. After cooling to room temperature, the mixture was diluted with dichloromethane, and the organic layer was separated and washed with deionized water and dried over anhydrous Na_2SO_4. The crude product was purified on a silica-gel column using dichloromethane: methanol (50:1 by volume, R_f = 0.2) as eluent. The desired product N-n-hexanoic acid-4-boronic acid pinacol ester-1,8-naphthimide (B-R-COOH) was obtained with 78% yield. ^{1}H NMR (400 MHz, DMSO-d_6) δ (ppm): 12.02 (s, 1H), 8.93 (d, J = 9.5 Hz, 1H), 8.38 (dd, J = 10.5, 7.3 Hz, 1H), 8.15 (d, J = 7.3 Hz, 1H), 7.86 – 7.80 (m, 1H), 4.01 – 3.95 (m, 1H), 2.22 (t, J = 7.2 Hz, 1H), 1.66 – 1.51 (m, 1H), 1.42 (s, 8H), 1.37 – 1.31 (m, 4H). ^{13}C NMR (101 MHz, DMSO-d_6) δ (ppm): 174.92, 163.62, 135.95, 134.81, 134.53, 130.75, 129.66, 127.82, 127.43 (s), 124.60, 122.59, 84.87, 73.97, 40.65, 34.00, 27.65, 26.52, 25.41, 25.17, 24.69. HRMS: $[M]^+$ Calcd. for 437.30; Found 438.21. The B-R-COONa probe was obtained by adjusting the pH of the B-R-COOH probe to 8.

2. Synthesis of the $NaYF_4$: Yb, Tm upconversion nanoparticles (UCNPs)

In a typical synthesis route[1], first, 0.6 g sodium hydroxide and 3 mL deionized water were mixed to form a clear and transparent solution, followed by adding 10 mL OA and 20 mL ethanol. 3.1 mL of 0.5 M $YCl_3·6H_2O$, 2.2 mL of 0.2 M $YbCl_3·6H_2O$, and 0.02 mL of 0.2 M $TmCl_3·6H_2O$ were added to a mixture by vigorous agitation for 20 min. Then, 2 mM NaF was then slowly added into the flask. After vigorous stirring at room temperature for 30 min, the colloidal solution was transferred into a 50 mL Teflon-lined autoclave, sealed and heated at 200 °C for 8 h. The systems were then allowed to naturally cool to room temperature, and thereafter the obtained products were washed with ethanol and cyclohexane, and then dried at 60 °C for 4 h.

3. Preparation of the test solutions and testing process

3.1. Preparation of the B-R-COOH probe solution

174.8 mg B-R-COOH probe was dissolved in 0.2 L of ethanol solution to get the B-R-COOH probe solution (2 mM). Then the solution was diluted with ethanol to obtain different concentrations of B-R-COOH probe solutions.

3.2. Preparation of the UCNPs solution

1.0000 g UCNPs was dissolved in 0.2 L of ethanol solution to get the UCNPs solution (5 mg/mL). Then the solution was diluted with ethanol to obtain different concentrations of

UCNPs solutions.

3.3. Preparation of the TBAH solution

TBAH (40% in water) stock solution was diluted with deionized water to obtain 40 mM, 20 mM, 10 mM, 6 mM, 2 mM, and 0 mM of TBAH solutions, respectively.

3.4. Preparation of the hydrogen peroxide (H_2O_2) solution

H_2O_2 (30%) stock solution was diluted with deionized water to obtain 12 mM, 10 mM, 8 mM, 7 mM, 6 mM, 5 mM, 4 mM, 3 mM, 2.5 mM, 2 mM, 1.8 mM, 1.6 mM, 1.4 mM, 1.0 mM, 0.8 mM, 0.6 mM, 0.4 mM, 0.2 mM, and 0 mM of H_2O_2 solutions, respectively.

3.5. Optimization of the H_2O_2 detection system

The main factors including system solvents, TBAH concentration, B-R-COOH probe concentration, and UCNPs concentration, which may greatly affect the detection performance, were optimized to gain a highly sensitive and reliable response. The ultraviolet-visible (UV-vis) absorption spectra, fluorescence spectra, and upconversion luminescence (UCL) spectra were used for monitoring the reaction product under different conditions.

Effect of solvents: Dissolved 31.5 mg of the B-R-COOH probe into 1.8 mL of methanol, ethanol, acetonitrile, acetone, tetrahydrofuran (THF), N, N-Dimethylformamide (DMF), and dimethyl sulfoxide (DMSO) solvent, respectively, and added 100 μL of TBAH solution (20 mM) and 100 μL of H_2O_2 solution (12 mM), the UV-vis absorbance and fluorescence intensity of the B-R-COOH probe were examined before and after adding H_2O_2 solution, respectively. As shown in Figure S11, the result illustrates that the optimal solvent environment determined by the change of UV-vis absorbance at 454 nm and fluorescence intensity at 558 nm before and after adding H_2O_2 solution were acetonitrile and ethanol, respectively. Considering that the fluorescence method usually has higher sensitivity than the colorimetric method, ethanol was chosen as the optimized solvent for the sensitive monitoring of H_2O_2.

Effect of TBAH concentration: In general, a catalyst has a great influence on the sensing performance. Using TBAH as the catalyst, the optimal ratio of C_{TBAH} to $C_{B\text{-}R\text{-}COOH}$ was investigated. The UV-vis absorption intensity at 454 nm and fluorescence intensity at 558 nm were examined with time after adding H_2O_2 solution, respectively. As shown in Figure S12, when C_{TBAH}: $C_{B\text{-}R\text{-}COOH}$ = 1:1, the best reaction rate for B-R-COOH probe with H_2O_2 was obtained. Therefore, C_{TBAH}: $C_{B\text{-}R\text{-}COOH}$ = 1:1 was chosen as the optimized TBAH concentration for the sensitive monitoring of H_2O_2.

Effect of B-R-COOH probe concentration: As illustrated in Figure S13, when the concentration of B-R-COOH probe in ethanol was fixed at 1 mM, and C_{TBAH}: $C_{B\text{-}R\text{-}COOH}$ = 1:1, the greatest change of the UV-vis absorption intensity at 454 nm and the fluorescence intensity

at 558 nm after adding H_2O_2 solution (600 μM) were observed. Therefore, 1 mM of B-R-COOH probe was selected as the optimized concentration for the sensitive monitoring of H_2O_2.

Effect of UCNPs concentration: As illustrated in Figure S20, when the concentration of UCNPs in ethanol was fixed at 0.5 mg/mL, the concentration of B-R-COOH at 1 mM, and C_{TBAH}: $C_{B\text{-}R\text{-}COOH}$ = 1:1, the maximum UCL quenching efficiency was observed after adding H_2O_2 solution (600 μM). The addition of UCNPs had almost no effect on the intensity of UV-vis absorption and fluorescence of B-R-COOH probe while detecting H_2O_2. Therefore, 0.5 mg/mL of UCNPs was selected as the optimal concentration for sensitive monitoring of H_2O_2.

3.6. Testing of H_2O_2 solution

Spectral response testing of the B-R-COOH probe: 100 μL of the H_2O_2 solution (0 mM, 0.2 mM, 0.4 mM, 0.6 mM, 0.8 mM, 1.0 mM, 1.4 mM, 1.6 mM, 1.8 mM, 2.0 mM, 2.5 mM, 3.0 mM, 4.0 mM, 5.0 mM, 6.0 mM, 7.0 mM, 8.0 mM, 10.0 mM, 12.0 mM, respectively) was added into the mixture solution of the TBAH solution (20 mM, 100 μL) and the B-R-COOH probe (1 mM, 1.8 mL), respectively. The obtained mixture solution was used for direct measurement of UV-vis absorption spectra (Spectrometer: Hitachi UV-3900), and fluorescence spectra (λex = 468 nm; Slits: 0.5 nm; λem = 558 nm; Slits: 0.5 nm; Spectrometer: Edinburgh FLS1000).

Spectral response testing of the UCNPs/B-R-COOH nanoprobe: 100 μL of the H_2O_2 solution (0 mM, 0.2 mM, 0.4 mM, 0.6 mM, 0.8 mM, 1.0 mM, 1.4 mM, 1.6 mM, 1.8 mM, 2.0 mM, 2.5 mM, 3.0 mM, 4.0 mM, 5.0 mM, 6.0 mM, 7.0 mM, 8.0 mM, 10.0 mM, 12.0 mM, respectively) was added into the mixture solution of the TBAH solution (20 mM, 100 μL), B-R-COOH probe (2 mM, 1 mL), and UCNPs (12.5 mg/mL, 0.8 mL), respectively. The obtained mixture solution was used for direct measurement of UV-vis absorption spectra (Spectrometer: Hitachi UV-3900), fluorescence spectra (λex = 468 nm; Slits: 0.5 nm; λem = 558 nm; Slits: 0.5 nm; Spectrometer: Edinburgh FLS1000), and UCL spectra (λex = 980 nm; λem = 475 nm; Slits: 0.5 nm; Spectrometer: Edinburgh FLS1000).

3.7. Preparation and testing of trace analytes

Analyte stock solution: $Na_2S_2O_8$, $NaBrO_3$, $NaNO_2$, KNO_3, $NaClO_4$, $KClO_3$, Urea, Sucrose, TNT, DNT, CH_3COONa, NaCl, NaOH, $MgCl_2$, NaF, KBr, $MgSO_4$, SDS, Detergent, Sanitizer, and Perfume were dissolved in deionized water to prepare the analyte stock solution with a concentration of 240 mM, respectively. Then, 240 mM of the analyte stock solutions were diluted one time with deionized water to obtain 120 mM analyte solutions.

Selectivity testing process: 100 μL of the analyte solution (120 mM) of certain analyte was added into a cuvette filled with the mixture solution of the TBAH solution (20 mM, 100 μL), B-R-COOH probe (2 mM, 1 mL), and UCNPs (12.5 mg/mL, 0.8 mL). The obtained mixture

solution was used for direct measurement of UV-vis absorption spectra (Spectrometer: Hitachi UV-3900), fluorescence spectra (λex = 468 nm; Slits: 0.5 nm; λem = 558 nm; Slits: 0.5 nm; Spectrometer: Edinburgh FLS1000), and UCL spectra (λex = 980 nm; λem = 475 nm; Slits: 0.5 nm; Spectrometer: Edinburgh FLS1000).

Anti-interference testing process: The mixture of the analyte solution of certain analyte (240 mM, 50 μL) and H_2O_2 solution (24 mM, 50 μL) was added into a cuvette filled with the mixture solution of the TBAH solution (20 mM, 100 μL), B-R-COOH probe (2 mM, 1 mL), and UCNPs (12.5 mg/mL, 0.8 mL). The obtained mixture solution was used for direct measurement of UV-vis absorption spectra (Spectrometer: Hitachi UV-3900), fluorescence spectra (λex = 468 nm; Slits: 0.5 nm; λem = 558 nm; Slits: 0.5 nm; Spectrometer: Edinburgh FLS1000), and UCL spectra (λex = 980 nm; λem = 475 nm; Slits: 0.5 nm; Spectrometer: Edinburgh FLS1000).

4. Preparation and testing of the UCNPs/B-R-COOH loaded sponge-based sensing chip for H_2O_2 vapor

4.1. Preparation of the UCNPs/B-R-COOH-loaded sponge sensing chip

A mixture solution of TBAH solution (20 mM, 2 μL), B-R-COOH probe solution (2 mM, 20 μL), and UCNPs solution (12.5 mg/mL, 16 μL) was added to a melamine sponge with an average pore size of 3×3×3 mm^3.

4.2. Preparation of H_2O_2 vapor

The H_2O_2 (30%) solution was diluted with deionized water to obtain different concentrations of H_2O_2 (0 mM, 12 mM, 24 mM, 49 mM, 98 mM, 196 mM, 326 mM, 490 mM, 979 mM, 1960 mM, and 4900 mM). 2 mL of the above mixture were taken into a conical flask and placed it in an oven at 40°C for 12 h to obtain H_2O_2 saturated vapor (0 ppm, 0.2 ppm, 0.4 ppm, 0.8 ppm, 1.6 ppm, 3.2 ppm, 5.3 ppm, 8.1 ppm, 16.4 ppm, 34.4 ppm, and 98.0 ppm).

4.3. Preparation of analyte vapors

2 mL of H_2O, methanol, acetone, THF, ethyl acetate, hexane, DMF, benzene, methylbenzene, coffee, tea, cooking oil, kerosene, perfume, and floral water solutions were taken separately in a conical flask and the respective saturated vapors were obtained by placing it in an oven at 40°C for 12 h.

4.4. Sensing testing of the sensing chip for H_2O_2 vapor

The sensing chips were placed in different concentrations of H_2O_2 vapor (0 ppm, 0.2 ppm, 0.4 ppm, 0.8 ppm, 1.6 ppm, 3.2 ppm, 5.3 ppm, 8.1 ppm, 16.4 ppm, 34.4 ppm, and 98.0 ppm) for 180 s. After that, the optical images were recorded with the smartphone under day light, 468 nm light and 980 nm light excitation.

4.5. Selectivity testing of the sensing chip for H_2O_2 vapor

The sensing chips were placed in the H_2O_2 vapor (98 ppm) and analyte vapors for 180 s, respectively. After that, the optical images were recorded with the smartphone under day light, 468 nm light and 980 nm light excitation.

4.6. Anti-interference testing of the sensing chip for H_2O_2 vapor

A gold pigment, a doderblue fluorescent powder, a red long afterglow material, the mixture of gold pigment and doderblue fluorescent powder, the mixture of gold pigment and red long afterglow material, and the mixture of doderblue fluorescent powder and red long afterglow material were placed on the sponge-based sensing chip respectively, and the corresponding images before and after adding H_2O_2 vapor were recorded with the smartphone under day light, 468 nm light and 980 nm light excitation.

5. Preparation and testing of the UCNPs/B-R-COOH loaded sponge-based sensing chip for triacetone triperoxide (TATP)

5.1. Preparation of TATP sample in the complicated environment

80 mg TATP was mixed with gold pigment, doderblue fluorescent powder, red long afterglow material, salt, cumin powder, chilli powder, coffee powder, milk-tea powder, amoxicillin powder, soil, and sand.

5.2. Sensing testing of the sensing chip for TATP sample

The TATP sample was added to a 20 ml sealed bottle, irradiated with a UV-vis light (λex = 365 nm) for 15 min, and the sensing chip was put into it. The corresponding optical images were observed with the smartphone under day light, 468 nm light and 980 nm light excitation.

6. Calculation Details

The geometries of the B-R-COOH probe, the resulting product N-n-caproate-4-hydroxy-1,8-naphthimide (H-R-COO$^-$) as well as the dimer of the probe or product were fully optimized using the CAM-B3LYP[2] functional combined with the def-2SVP[3] basis set, and the dispersion correction term (D3)[4] was included to improve the accuracy of the calculations.

All of the calculation was performed based on the density functional theory (DFT), including the solvent effect using the solvation model based on density (SMD)[5] solvation model with self-defined parameter (eps = 29.9 and epsinf = 1.97872), which obtained from the experimental mixed-solvent environment.

Based on it, the Gibbs free energy difference of the ionic dissociation process of the B-R-COOH probe was calculated in the context of fully considering the thermodynamic factor using the equation as

$$\Delta G_{aq}^{1M} = G_{gas}(Probe^-) - G_{gas}(Probe) + \Delta G_{solv}^{mod}(Probe^-) - \Delta G_{solv}^{mod}(Probe) - 270.29$$

*which the $G_{gas}(Probe^{-})$, $G_{gas}(Probe)$, $\Delta G_{solv}^{mod}(Probe^{-})$ and $\Delta G_{solv}^{mod}(Probe)$ represented as the Gibbs free energy of the B-R-COO^{-} and B-R-COOH, the dissolution free energy of B-R-COO^{-} and B-R-COOH without the standard state.

The Gibbs free energy calculations were performed at the CAM-B3LYP/def2-TZVPP level with the frequency calculations to ensure that the systems represent true minima on the PES, and to provide corrections for the zero-point vibrational energy (ZPVE)[6] effects in Shermo software[7].

In order to simulate the UV-vis absorption and fluorescence emission condition, the time dependent density functional theory (TD-DFT)[8] calculations were carried out to obtain the vertical excitation energies for 40 lowest singlet transitions and the first excited state optimized geometry at TPSSh[9]/def2-SVPP level. The convolution of the spectrum was obtained using a gaussian function with a full width at half maximum (FWHM) of 0.3 eV.

The molecular dynamics (MD) simulations were performed using the GROMACS package[10] with the computational model constituted by the initial configurations of 1 B-R-COOH probe, 308 ethylene glycol, 202 H_2O and 150 H_2O_2 molecules. The generalized OPLS-AA force field parameters were used to describe bonded and nonbonded interactions for all studied molecules[11]. The restrained electrostatic potential (RESP) charges calculated at the CAM-B3LYP/def2-TZVPP[3] with the Grimme's DFT-D3(BJ) level were used to describe electrostatic properties of all studied molecules. Gaussian 09 was used to optimize molecular structure and obtain wave function. RESP charges were calculated by Multiwfn[12]. The initial configurations for MD production runs were randomly assembled through PACKMOL package[13]. The 20 ns normal pressure and temperature (NPT) simulation with a Velocity-rescale thermostat, and Parrinello-Rahman barostat[14] with the time constants of couplings being 0.2 and 2.0 ps of production of MD simulation was performed to obtain the final equilibrium state. When calculating the interaction between molecules, the electrostatic and Lennard-Jones interactions were calculated by setting with a cut-off of 2.5 nm. Periodic boundary conditions were applied in all three dimensions.

The Hirshfeld surface analysis[15] was performed to quantitatively reveal the weak interactions between the B-R-COOH probe and the surrounding H_2O_2 molecules through the ratio of the electron distribution on the molecular surface under promolecular approximation cartography.

In which, the normalized contact distance (d_{norm}) could be decomposed according the equation:

$$d_{norm} = \frac{d_i - r_i^{vdW}}{r_i^{vdW}} - \frac{d_e - r_e^{vdW}}{r_e^{vdW}}$$

when where d_i and r_i^{vdW} (d_e and r_e^{vdW}) are the distance from a point on the surface to the nearest nucleus inside (outside) the surface and vdW radius of the corresponding two atoms.

The d_{norm} parameters were used to map the 3D Hirshfeld surfaces of the H_2O_2 molecules surrounding the B-R-COOH probe with a color scheme in a range of blue, white, and red, which represent no interaction, weak attraction, and strong attraction, respectively.

Furthermore, the combination of d_i and d_e in the form of a 2D fingerprint plot could represents a summary of intermolecular contacts between the B-R-COOH probe and the surrounding H_2O_2 on the Hirshfeld surfaces.

The Quickstep module of the CP2K software[16, 17] employing PBE0[18] functional combined the mixed Gaussian and plane wave basis set (DZVP-MOLOPT-SR-GTH)[19, 20] was performed to investigate the electrostatic binding between the UCNPs and B-R-COOH probe based on the ab initio theory. It should be noted that during the structural optimization process, the orbital transformation (OT) method was applied and the SCF convergence target was set to 5×10^{-6} a.u. with a plane wave cutoff of 400 Ry. Firstly, the foundational $NaYF_4$ cell was optimized by using (3,3,3) Monkhorst-Pack grids without the OT method, then a seven-layered $NaYF_4$ model with the exposed (100) facet was created from the optimized $NaYF_4$ cell. To prevent interaction between adjacent periodic systems, a 40 Å vacuum layer was added. The bottom four layers of the $NaYF_4$ model were kept fixed, while all other atoms were allowed to undergo normal relaxation. After further optimizing the conformation of the $NaYF_4$ model, the B-R-COOH molecule was positioned into the model with its carboxyl tail near the surface of $NaYF_4$. By structure optimization with a cutoff energy of 400 Ry, the single-point energies were calculated for $NaYF_4$, B-R-COOH, and the $NaYF_4$ (100)/B-R-COOH system with the electronic energies of these three systems labelling as E_{NaYF_4}, $E_{B-R-COOH}$, and $E_{NaYF_4(100)/B-R-COOH}$, respectively. The simultaneously generated cube files were utilized for the electron density difference (EDD) analysis, and the binding energy (E_{BE}) between the UCNPs and B-R-COOH probe could be determined by the equation: $E_{BE} = E_{NaYF_4(100)/B-R-COOH} - E_{B-R-COOH} - E_{NaYF_4}$.

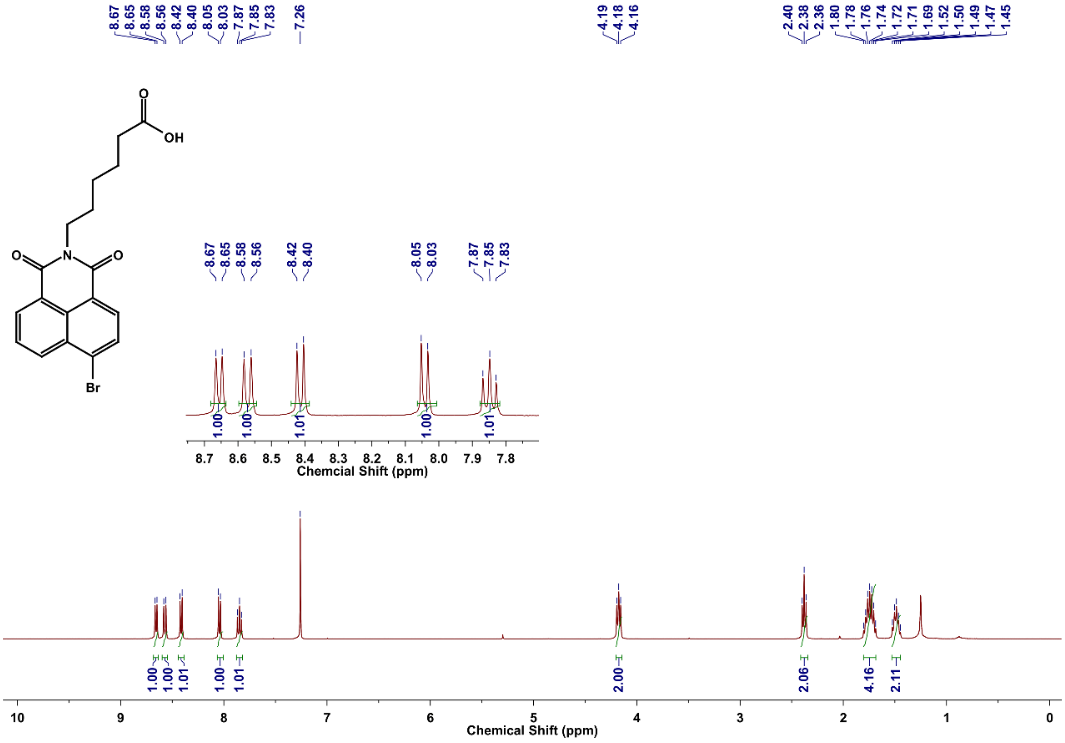

Figure S1. ^{1}H NMR spectrum of 1 ($CDCl_3$, 400 MHz).

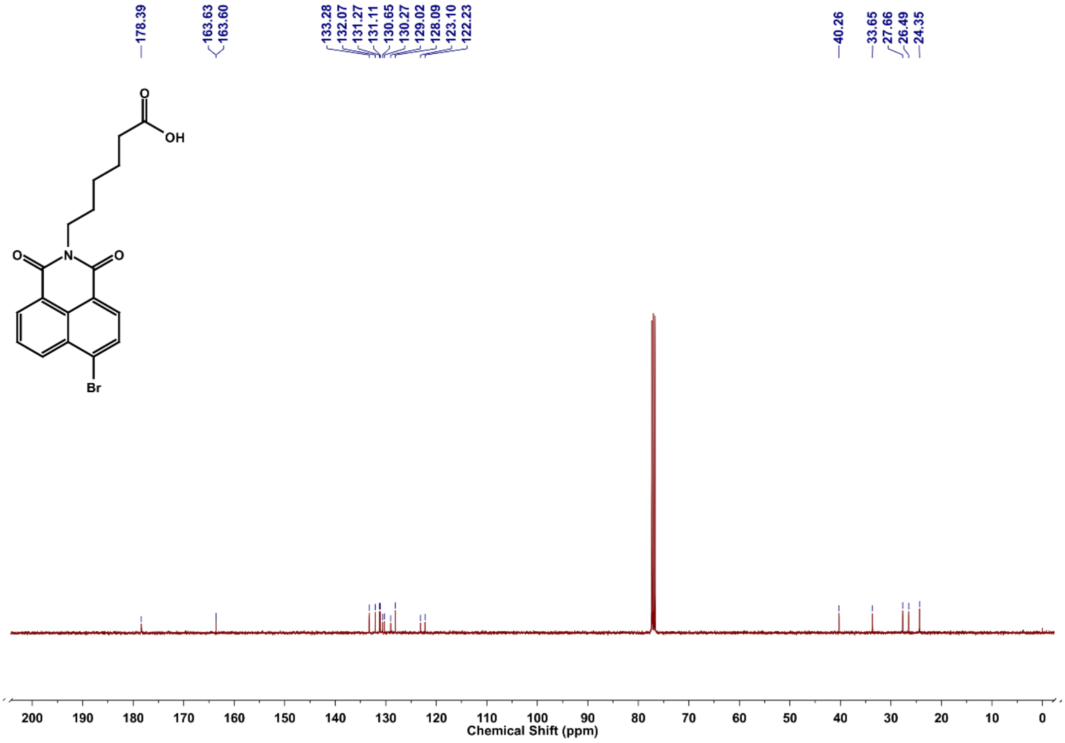

Figure S2. ^{13}C NMR spectrum of 1 ($CDCl_3$, 101 MHz).

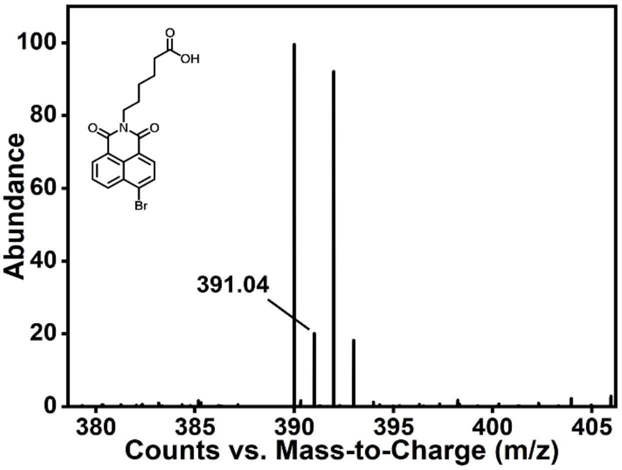

Figure S3. HRMS spectrum of **1**.

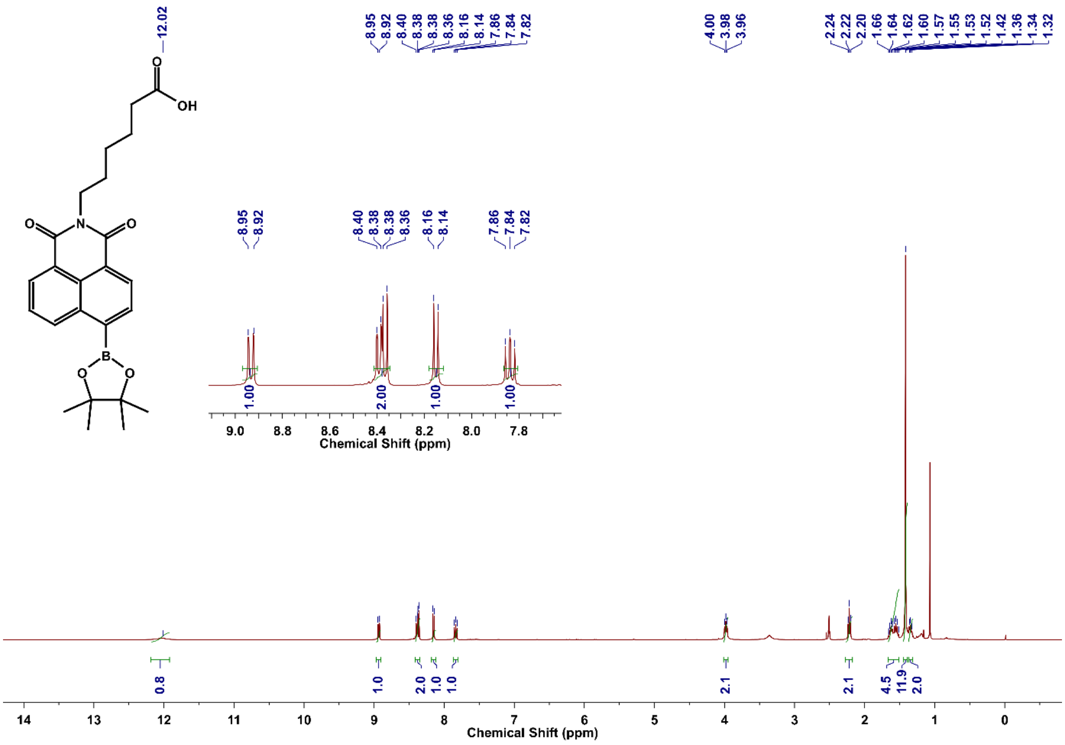

Figure S4. ^{1}H NMR spectrum of the B-R-COOH probe (DMSO-d_6, 400 MHz).

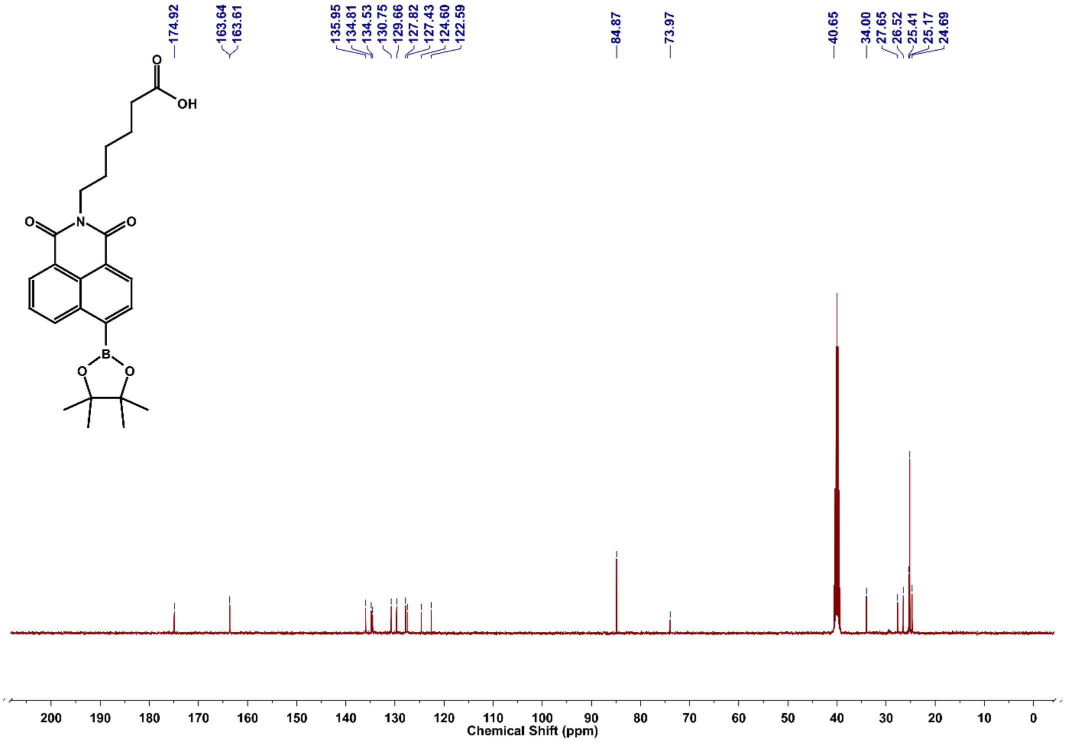

Figure S5. ^{13}C NMR spectrum of the B-R-COOH probe (DMSO-d_6, 101 MHz).

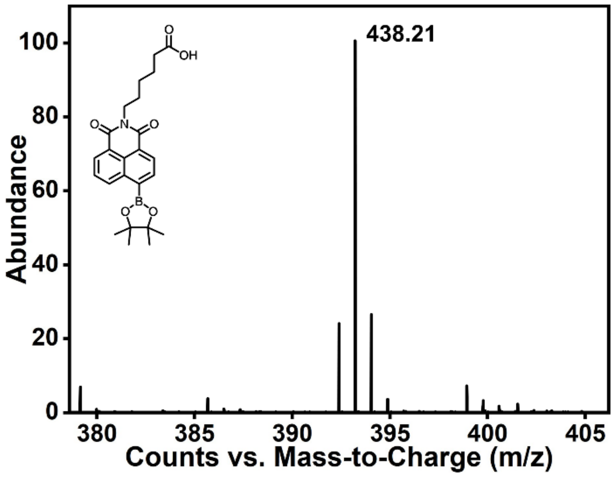

Figure S6. HRMS spectrum of the B-R-COOH probe.

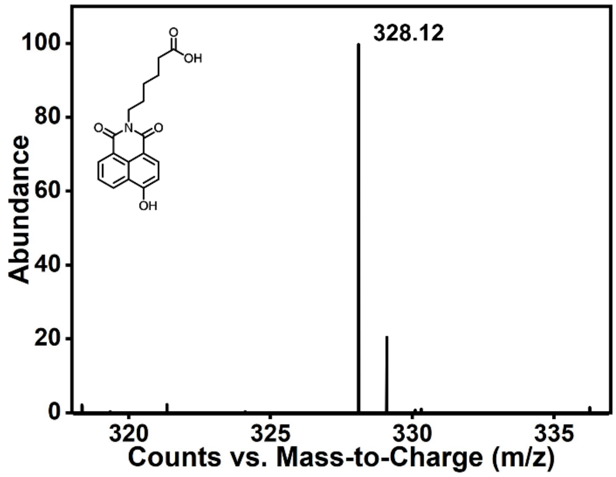

Figure S7. HRMS spectrum of the H-R-COOH product.

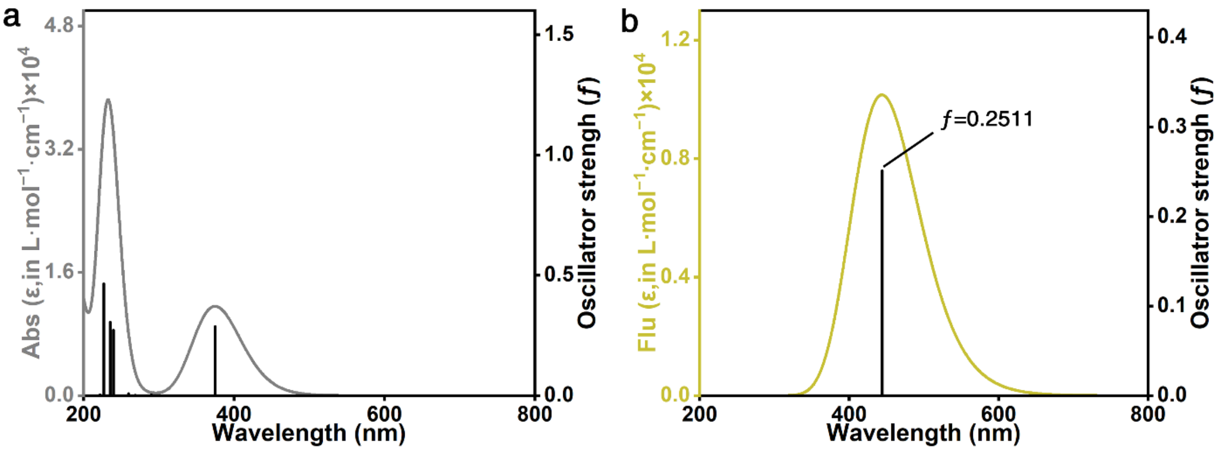

Figure S8. Simulated (a) UV-vis absorption spectra and (b) fluorescence spectra (curve), and the oscillator strength (spikes) of the H-R-COO$^-$ monomer.

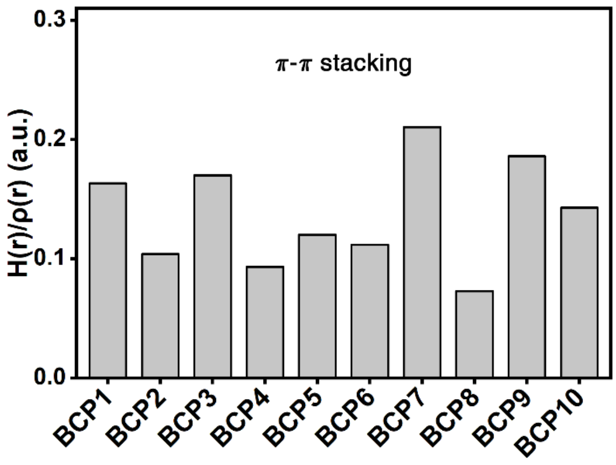

Figure S9. The H(r)/ρ(r) values of the intermolecular interactions of the H-R-COO$^-$ dimer.

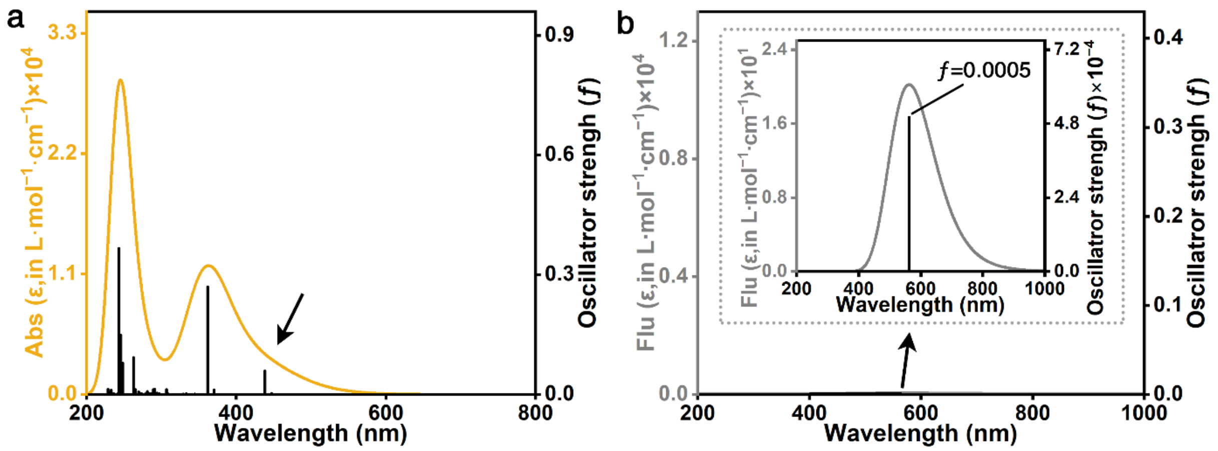

Figure S10. Simulated (a) UV-vis absorption spectra and (b) fluorescence spectra (curve), and the oscillator strength (spikes) of the H-R-COO$^-$ dimer.

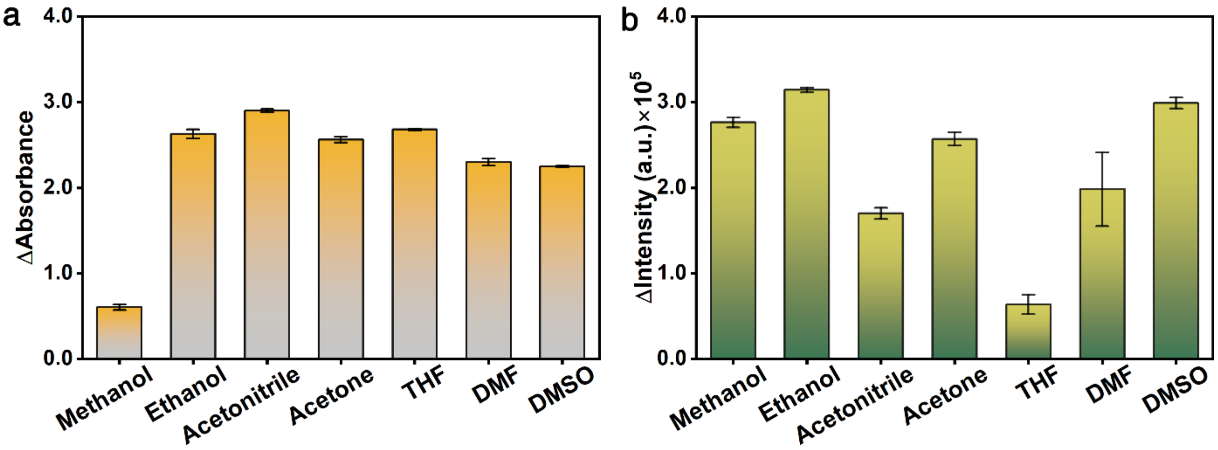

Figure S11. (a) UV-vis absorption intensity variations at 454 nm and (b) fluorescence intensity variations at 558 nm of the B-R-COOH probe in different solvents before and after adding H_2O_2.

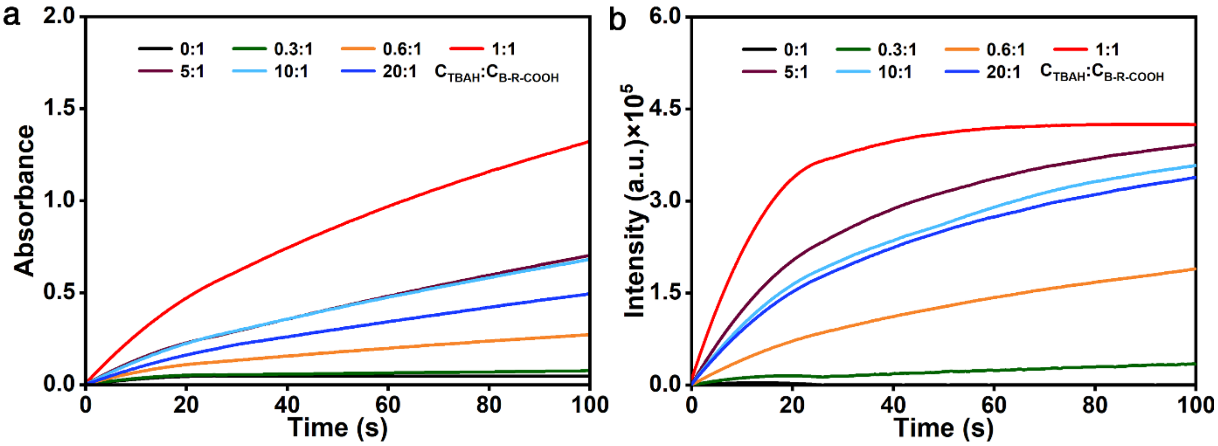

Figure S12. The time-dependent (a) UV-vis absorption (454 nm) response and (b) fluorescent (558 nm) response of the B-R-COOH probe with different C_{TBAH}: $C_{B\text{-}R\text{-}COOH}$ for detection H_2O_2.

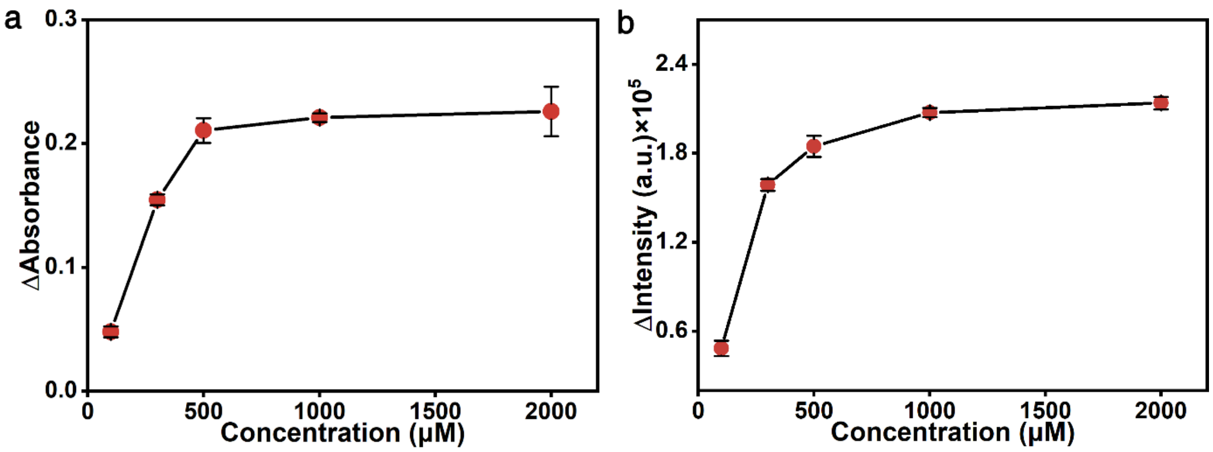

Figure S13. The influence of the B-R-COOH probe concentration on the (a) UV-vis absorption intensity variations at 454 nm and (b) fluorescence intensity variations at 558 nm before and after adding H_2O_2.

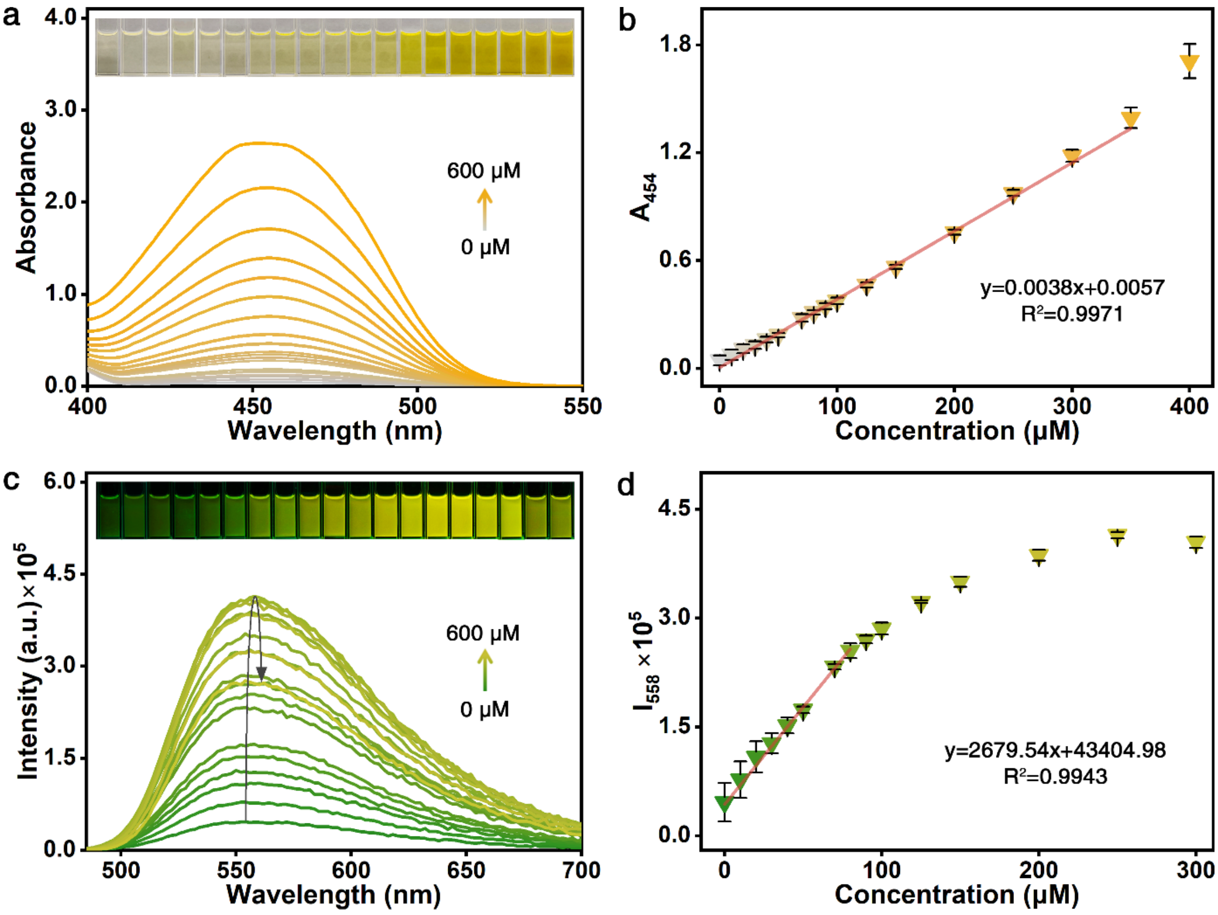

Figure S14. The optical spectra and the corresponding images (inset) from B-R-COOH probe towards H_2O_2 under (a) colorimetric and (c) fluorescent mode. The corresponding (b) absorption intensity (A_{454}) and (d) fluorescence intensity (I_{558}) as a function of H_2O_2 concentration.

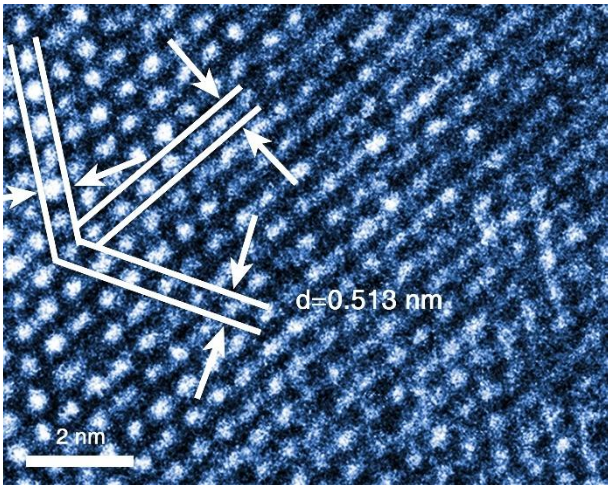

Figure S15. HRTEM image of the UCNPs.

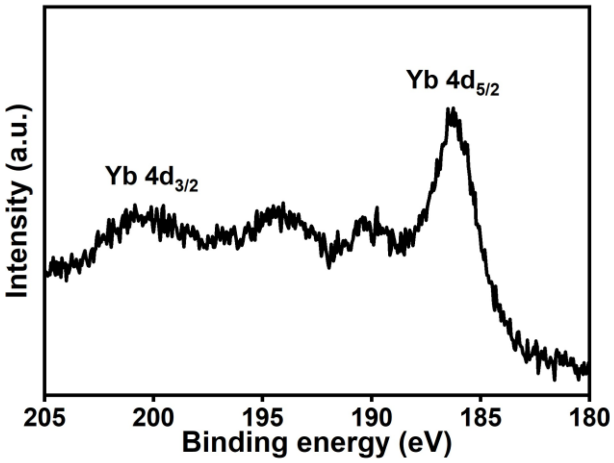

Figure S16. XPS spectra of the Yb 4d region of the UCNPs.

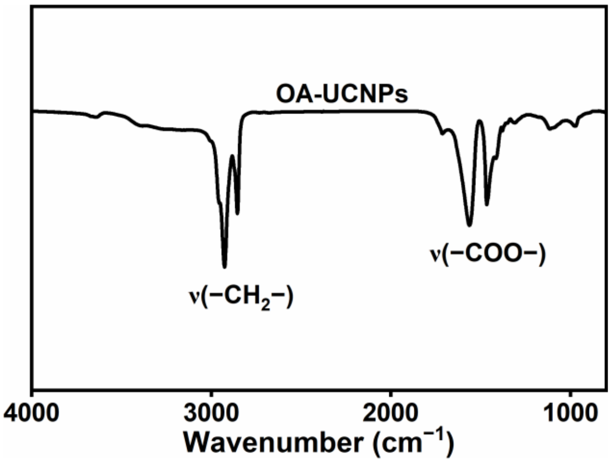

Figure S17. FT-IR spectra of the UCNPs.

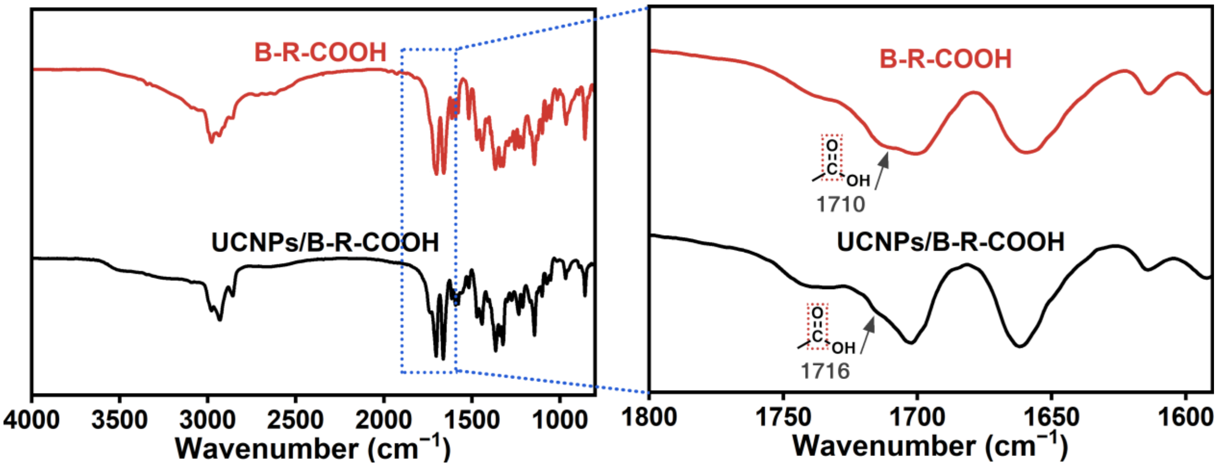

Figure S18. FT-IR spectra of the B-R-COOH probe and UCNPs/B-R-COOH nanoprobe and the enlarged FT-IR spectra from 1800 cm^{-1} to 1590 cm^{-1}.

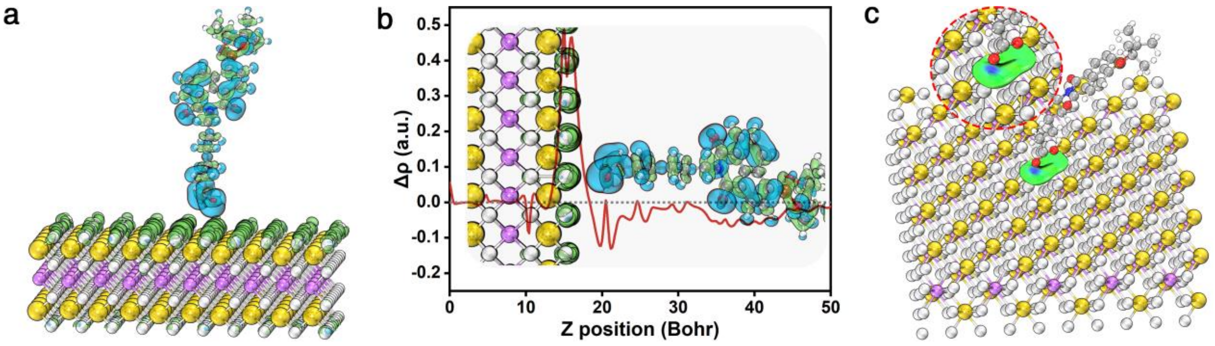

Figure S19. (a) The EDD distributions of the interface between the B-R-COOH probe and $NaYF_4$ (100) UCNPs. (b) The charge displacement curves of the $NaYF_4$ (100)/B-R-COOH. (c) The IGM analysis of the interactions between the B-R-COOH probe and $NaYF_4$ (100) UCNPs.

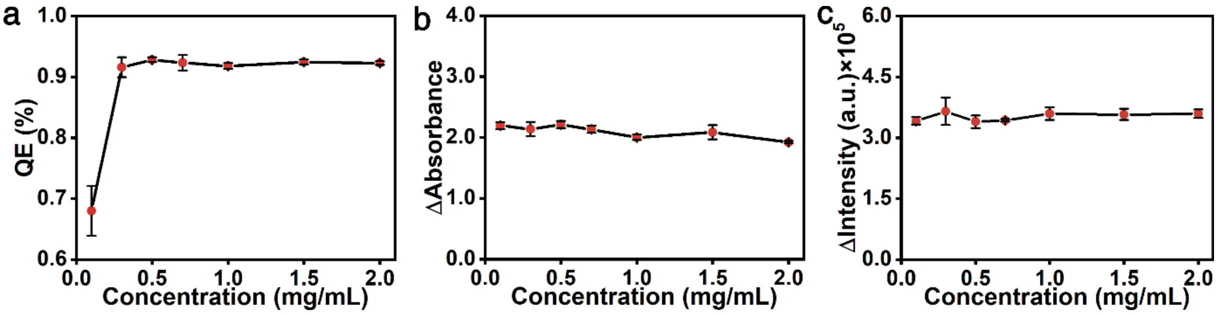

Figure S20. The influence of UCNPs concentration on the (a) UCL quenching efficiency, (b) UV-vis absorption intensity variations at 454 nm, and (c) fluorescence intensity variations at 558 nm of the UCNPs/B-R-COOH nanoprobe for detecting H_2O_2.

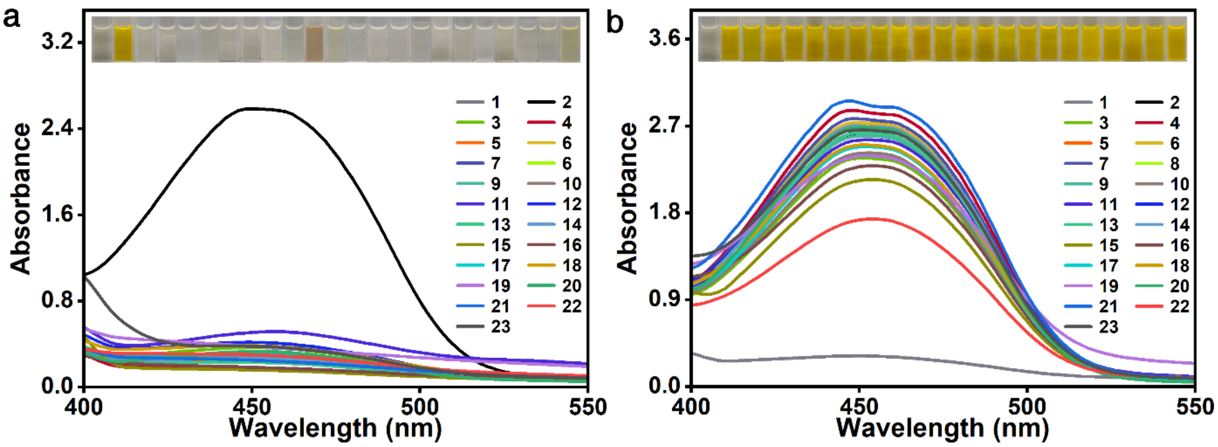

Figure S21. Selectivity and anti-interference of the UCNPs/B-R-COOH nanoprobe toward H_2O_2 in the colorimetric mode. UV-vis absorption spectra and images (inset) of the UCNPs/B-R-COOH nanoprobe in the presence of (a) H_2O_2 (600 μM) or various interferences (6 mM), and (b) the mixture solution of various interferences (6 mM) and H_2O_2 (600 μM). 1: Blank, 2: H_2O_2, 3: $Na_2S_2O_8$, 4: $NaBrO_3$, 5: $NaNO_2$, 6: KNO_3, 7: $NaClO_4$, 8: $KClO_3$, 9: Urea, 10: Sucrose, 11: TNT, 12: DNT, 13: CH_3COONa, 14: NaCl, 15: NaOH, 16: $MgCl_2$, 17: NaF, 18: KBr, 19: $MgSO_4$, 20: SDS, 21: Detergent, 22: Hand sanitizer, 23: Perfume.

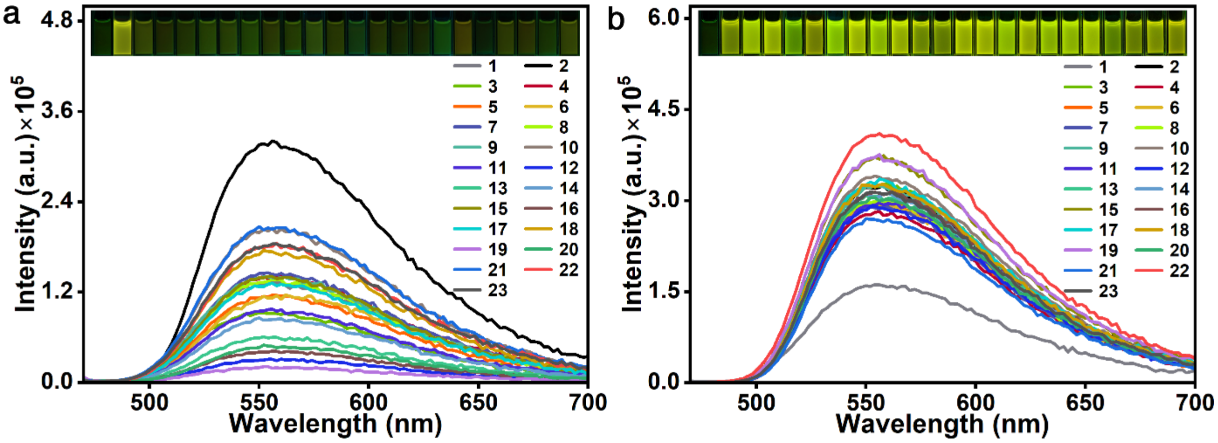

Figure S22. Selectivity and anti-interference of the UCNPs/B-R-COOH nanoprobe toward H_2O_2 in the fluorescent mode. Fluorescence emission spectra and images (inset) of the UCNPs/B-R-COOH nanoprobe in the presence of (a) H_2O_2 (600 μM) or various interferences (6 mM), and (b) the mixture solution of various interferences (6 mM) and H_2O_2 (600 μM). 1: Blank, 2: H_2O_2, 3: $Na_2S_2O_8$, 4: $NaBrO_3$, 5: $NaNO_2$, 6: KNO_3, 7: $NaClO_4$, 8: $KClO_3$, 9: Urea, 10: Sucrose, 11: TNT, 12: DNT, 13: CH_3COONa, 14: NaCl, 15: NaOH, 16: $MgCl_2$, 17: NaF, 18: KBr, 19: $MgSO_4$, 20: SDS, 21: Detergent, 22: Hand sanitizer, 23: Perfume.

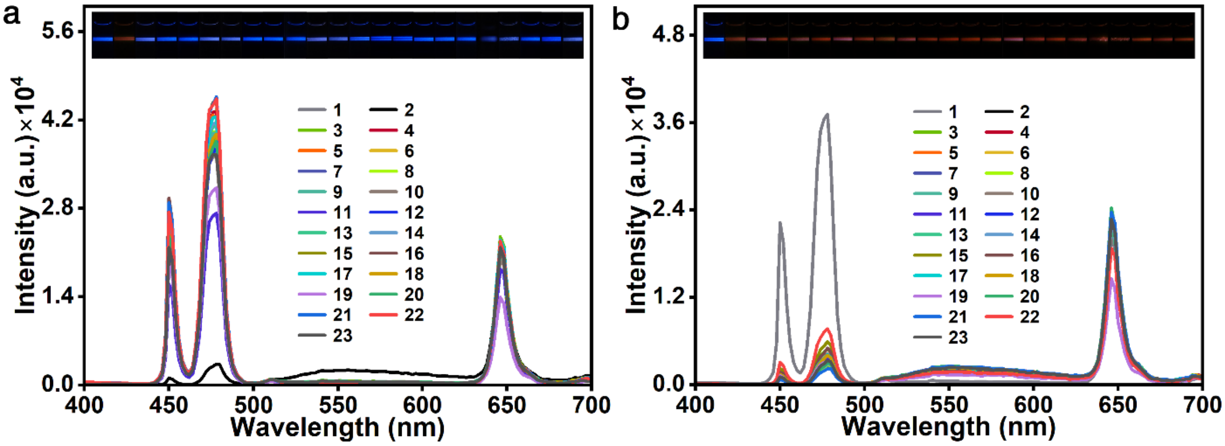

Figure S23. Selectivity and anti-interference of the UCNPs/B-R-COOH nanoprobe toward H_2O_2. in the UCL mode. UCL spectra and images (inset) of the UCNPs/B-R-COOH nanoprobe in the presence of (a) H_2O_2 (600 μM) or various interferences (6 mM), and (b) the mixture solution of various interferences (6 mM) and H_2O_2 (600 μM). 1: Blank, 2: H_2O_2, 3: $Na_2S_2O_8$, 4: $NaBrO_3$, 5: $NaNO_2$, 6: KNO_3, 7: $NaClO_4$, 8: $KClO_3$, 9: Urea, 10: Sucrose, 11: TNT, 12: DNT, 13: CH_3COONa, 14: NaCl, 15: NaOH, 16: $MgCl_2$, 17: NaF, 18: KBr, 19: $MgSO_4$, 20: SDS, 21: Detergent, 22: Hand sanitizer, 23: Perfume.

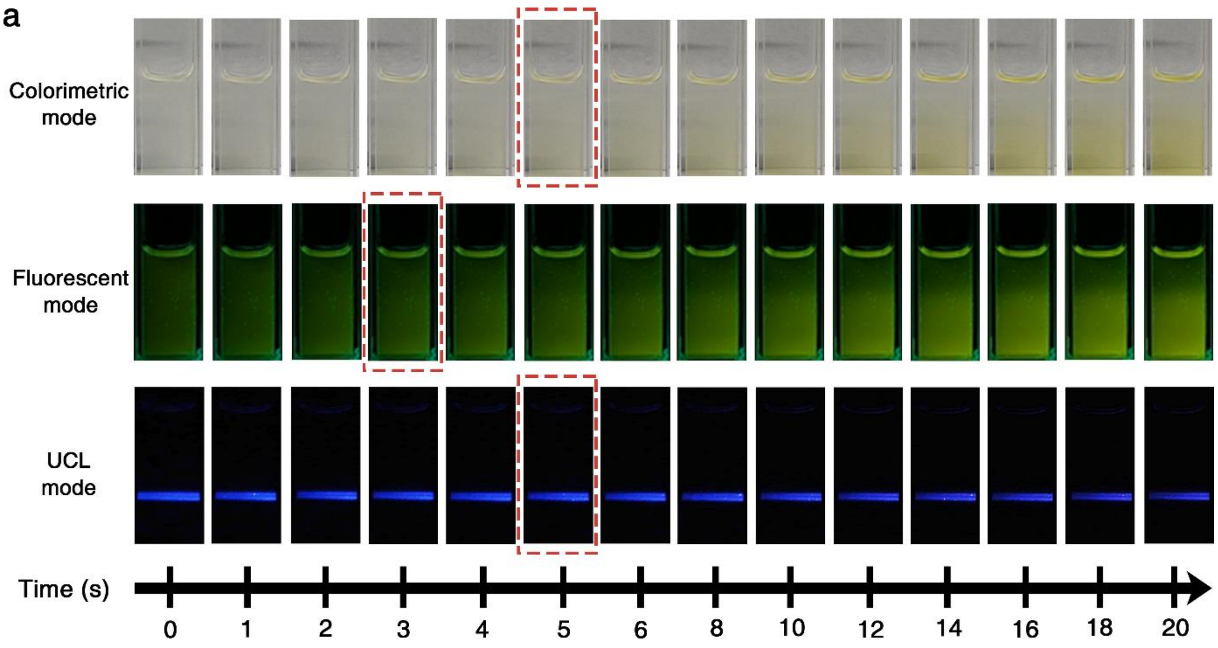

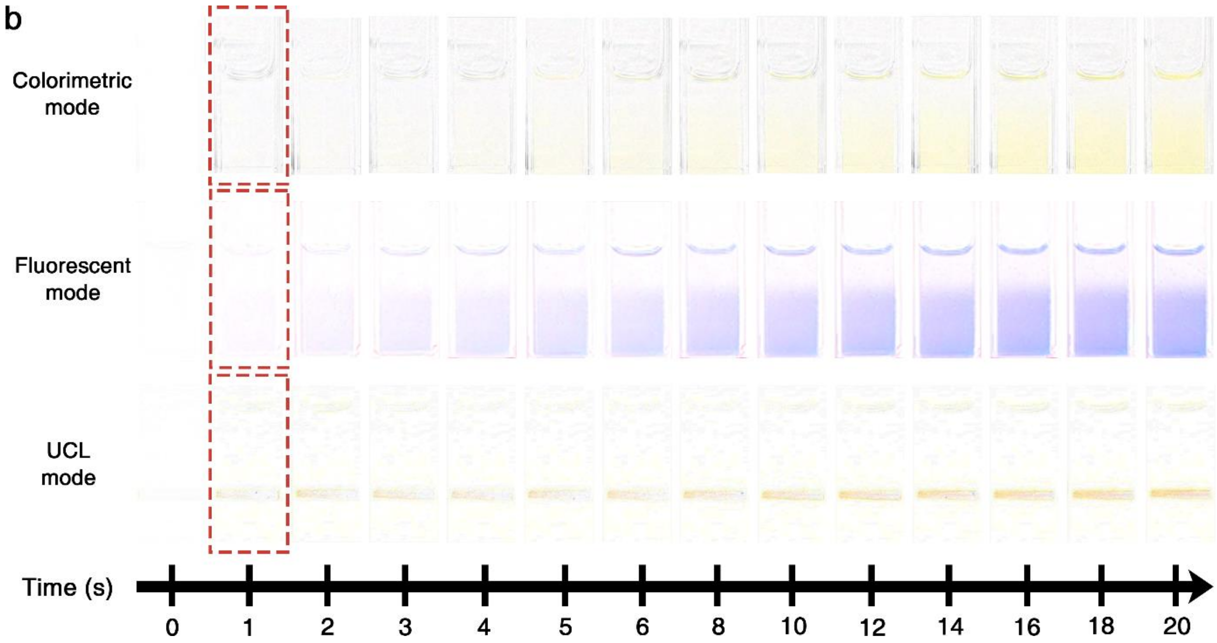

Figure S24. The real-time response of UCNPs/B-R-COOH nanoprobe for detecting H_2O_2 under colorimetric, fluorescent and UCL mode. (a) Optical images and (b) corresponding difference images.

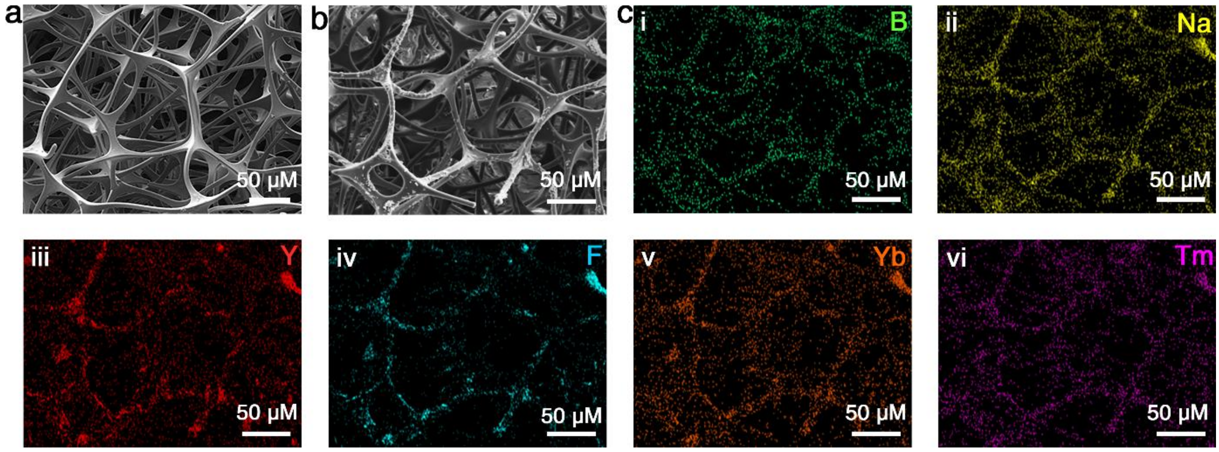

Figure S25. SEM images of (a) sponge and (b) the UCNPs/B-R-COOH nanoprobe loaded sponge. (c) The corresponding EDS mappings of (i) B, (ii) Na, (iii) Y, (iv) F, (v) Yb, (vi) Tm element.

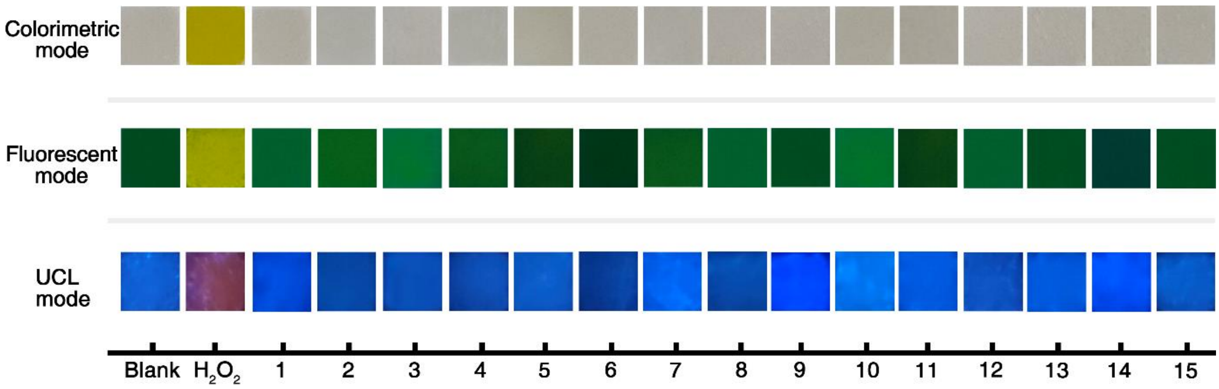

Figure S26. Optical images of the selectivity characterization of the sensing chip towards H_2O_2 and other 15 types of interferents vapor under colorimetric, fluorescent and UCL mode. 1: H_2O, 2: Methanol, 3: Acetone, 4: THF, 5: Ethyl acetate, 6: Hexane, 7: DMF, 8: Benzene, 9: Methylbenzene, 10: Coffee, 11: Tea, 12: Cooking oil, 13: Kerosene, 14: Perfume, 15: Floral water.

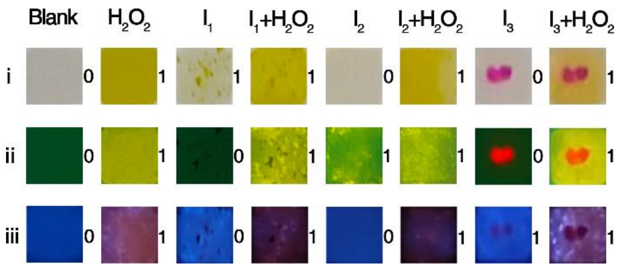

Figure S27. Optical images of the anti-interference capability characterization of the sensing chip towards H_2O_2, and the mixture of H_2O_2 and interferences. I_1: gold pigment, I_2: doderblue fluorescent powder, I_3: red long afterglow material.

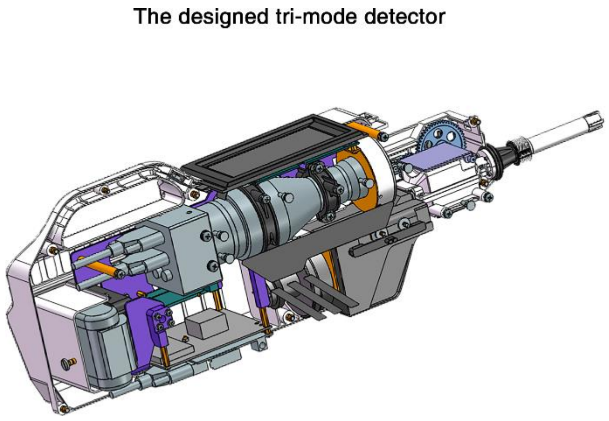

Figure S28. Schematic illumination of the designed tri-mode portable detector.

Table S1. The binding energy of the B-R-COOH probe dimer and H-R-COO⁻ product dimer.

Name	Energy (hartees)	Binding energy (hartees)	Binding energy (kcal/mol)
B-R-COOH probe dimer	−2925.21	−0.0089	−5.60
B-R-COOH probe monomer 1	−1462.60	/	/
B-R-COOH probe monomer 2	−1462.60	/	/
H-R-COO⁻ product dimer	−2254.32	−0.0215	−13.47
H-R-COO⁻ product monomer 1	−1127.15	/	/
H-R-COO⁻ product monomer 2	−1127.15	/	/

Table S2. Comparison of recently developed colorimetric or fluorescent methods for H_2O_2 detection.

Probe	Method	Linear range	Response time	LOD	Anti-interference	Ref.
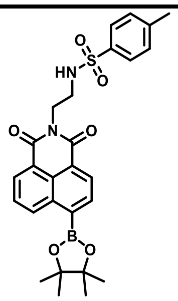	Colorimetry<br>Fluorometry	/<br>0–40 μM	/<br>/	/<br>120 nM	/	[21]
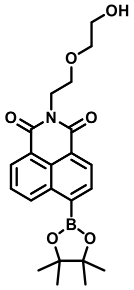	Colorimetry<br>Fluorometry	0–140 μM<br>0–180 μM	/<br>40 min	/<br>2 μM	Oxidants, Metallic salts	[22]
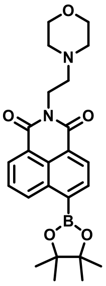	Colorimetry<br>Fluorometry	/<br>1–20 μM	/<br>60 s	/<br>1.21 μM	/	[23]

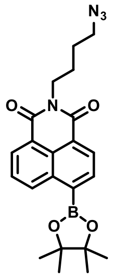	Fluorometry	1–10 μM	/	0.17 μM	/	[24]
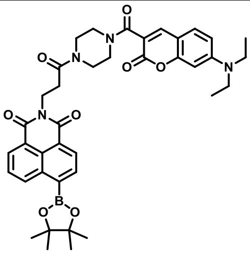	Colorimetry<br>Fluorometry	/<br>0–60 μM	/<br>/	/<br>0.28 μM	/	[25]
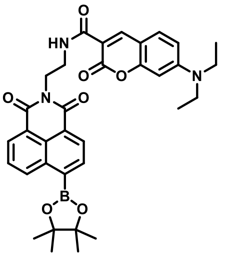	Colorimetry<br>Fluorometry	/<br>30–200 μM	/<br>60 min	/<br>1.35 μM	/	[26]
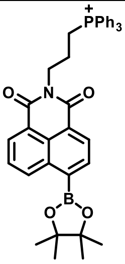	Colorimetry<br>Fluorometry	/<br>0–30 μM	/<br>60 min	/<br>0.58μM	/	[27]
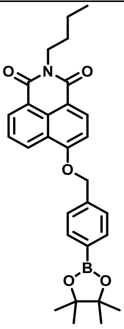	Colorimetry<br>Fluorometry	18–540 μM<br>18–540 μM	/<br>10 min	50 μM<br>4 μM	Oxidants, Metallic salts	[28]
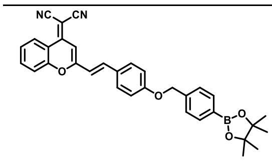	Colorimetry<br>Fluorometry	/<br>0.08–18 μM	/<br>30 min	/<br>70 nM	Oxidants, Metallic salts, Daily compounds	[29]
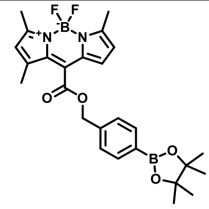	Colorimetry<br>Fluorometry	/<br>0–50 μM	/<br>/	/<br>0.15 μM	/	[30]

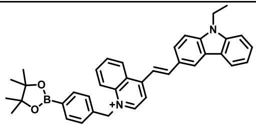	Colorimetry<br>Fluorometry	/<br>0.2–10 μM	/<br>4 min	/<br>40 nM	/	[31]
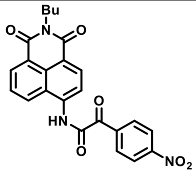	Colorimetry<br>Fluorometry	/<br>0–1 μM	/<br>/	/<br>37 nM	Oxidants, Metallic salts, Daily compounds	[32]
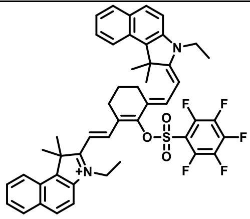	Colorimetry<br>Fluorometry	/<br>0–100 μM	/<br>200 s	/<br>50 nM	/	[33]
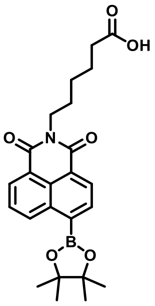	**Colorimetry**<br>**Fluorometry**	**0–350 μM**<br>**0–80 μM**	**1 s**<br>**1 s**	**236.84 nM**<br>**3.36 nM**	/	**This work**
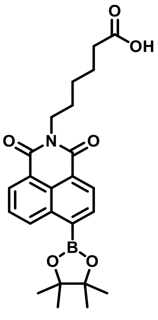<br>**+$NaYF_4$: Yb, Tm**	**Colorimetry**<br>**Fluorometry**<br>**UCL**	**0–125 μM**<br>**0–70 μM**<br>**0–200 μM**	**1 s**<br>**1 s**<br>**1 s**	**375.00 nM**<br>**4.34 nM**<br>**94.36 nM**	**Oxidants, Explosives, Metallic salts, Daily compounds, Colorful and fluorescent substances**	**This work**

Table S3. The binding energy of the B-R-COOH probe and $NaYF_4$ (100) UCNPs.

**Name**	**Total energy (hartees)**	**Binding energy (hartees)**	**Binding energy (eV)**
$NaYF_4$ (100)/B-R-COOH	−16187.35	−0.10	−2.77
$NaYF_4$ (100)	−15925.02	/	/
B-R-COOH	−262.23	/	/

References

[1] Z. Lyu, H. Dong, X. Yang, L. Huang, Y. Xu, K. Wu, L. Sun, C. Yan, *JACS Au* **2023**, *3*,

860.
[2] T. Yanai, D. P. Tew, N. C. Handy, *Chem. Phys. Lett.* **2004**, *393*, 51.
[3] A. Schäfer, H. Horn, R. Ahlrichs, *J. Chem. Phys.* **1992**, *97*, 2571.
[4] S. Grimme, J. Antony, S. Ehrlich, H. Krieg, *J. Chem. Phys.* **2010**, *132*, 154104.
[5] A. V. Marenich, C. J. Cramer, D. G. Truhlar, *J. Phys. Chem. B* **2009**, *113*, 6378.
[6] I. M. Alecu, J. Zheng, Y. Zhao, D. G. Truhlar, *J. Chem. Theory Comput.* **2010**, *6*, 2872.
[7] T. Lu, Q. Chen, *Comput. Theor. Chem.* **2021**, *1200*, 113249.
[8] G. Scalmani, M. J. Frisch, B. Mennucci, J. Tomasi, R. Cammi, V. Barone, *J. Chem. Phys.* **2006**, *124*, 094107.
[9] J. Tao, J. P. Perdew, V. N. Staroverov, G. E. Scuseria, *Phys. Rev. Lett.* **2003**, *91*, 146401.
[10] H. J. C. Berendsen, D. van der Spoel, R. van Drunen, *Comput. Phys. Commun.* **1995**, *91*, 43.
[11] W. L. Jorgensen, J. Chandrasekhar, J. D. Madura, R. W. Impey, M. L. Klein, *J. Chem. Phys.* **1983**, *79*, 926.
[12] T. Lu, F. W. Chen, *J.Comput. Chem.* **2012**, *33*, 580.
[13] L. Martinez, R. Andrade, E. G. Birgin, J. M. Martinez, *J. Comput. Chem.* **2009**, *30*, 2157.
[14] M. Parrinello, A. Rahman, *J. Appl. Phys.* **1981**, *52*, 7182.
[15] M. A. Spackman, D. Jayatilaka, *CrystEngComm* **2009**, *11*, 19.
[16] J. VandeVondele, M. Krack, F. Mohamed, M. Parrinello, T. Chassaing, J. Hutter, *Comput. Phys. Commun.* **2005**, *167*, 103.
[17] T. Kühne, M. Iannuzzi, M. Del Ben, V. Rybkin, P. Seewald, F. Stein, T. Laino, R. Khaliullin, O. Schütt, F. Schiffmann, D. Golze, J. Wilhelm, S. Chulkov, M. Bani-Hashemian, V. Weber, U. Borštnik, M. Taillefumier, A. Jakobovits, A. Lazzaro, H. Pabst, T. Müller, R. Schade, M. Guidon, S. Andermatt, N. Holmberg, G. Schenter, A. Hehn, A. Bussy, F. Belleflamme, G. Tabacchi, A. Glöß, M. Lass, I. Bethune, C. Mundy, C. Plessl, M. Watkins, J. VandeVondele, M. Krack, J. Hutter, *J. Chem. Phys.* **2020**, *152*, 194103.
[18] J. P. Perdew, K. Burke, M. Ernzerhof, *Phys. Rev. Lett.* **1996**, *77*, 3865.
[19] C. Hartwigsen, S. Goedecker, J. Hutter, *Phys. Rev. B* **1998**, *58*, 3641.
[20] S. Goedecker, M. Teter, J. Hutter, *Phys. Rev. B* **1996**, *54*, 1703.
[21] H. Xiao, P. Li, X. Hu, X. Shi, W. Zhang, B. Tang, *Chem. Sci.* **2016**, *7*, 6153.
[22] B. Zhu, H. Jiang, B. Guo, C. Shao, H. Wu, B. Du, Q. Wei, *Sens. Actuators B Chem.* **2013**, *186*, 681.
[23] M. Ren, B. Deng, J. Wang, X. Kong, Z. Liu, K. Zhou, L. He, W. Lin, *Biosens. Bioelectron.* **2016**, *79*, 237.

[24] Y. Wen, K. Liu, H. Yang, Y. Li, H. Lan, Y. Liu, X. Zhang, T. Yi, *Anal. Chem.* **2014**, *86*, 9970.

[25] X. Liang, X. Xu, D. Qiao, Z. Yin, L. Shang, *Chem. Asian J.* **2017**, *12*, 3187.

[26] K. Xu, L. He, X. Yang, Y. Yang, W. Lin, *Analyst* **2018**, *143*, 3555.

[27] F. Dai, F. Jin, Y. Long, X. Jin, B. Zhou, *Free Radical Res.* **2018**, *52*, 1288.

[28] C. Liu, C. Shao, H. Wu, B. Guo, B. Zhu, X. Zhang, *RSC Adv.* **2014**, *4*, 16055.

[29] Z. Zhou, Y. Li, W. Su, B. Gu, H. Xu, C. Wu, P. Yin, H. Li, Y. Zhang, *Sens. Actuators B Chem.* **2019**, *280*, 120.

[30] H. Chen, X. He, M. Su, W. Zhai, H. Zhang, C. Li, *J. Am. Chem. Soc.* **2017**, *139*, 10157.

[31] J. Xu, Y. Zhang, H. Yu, X. Gao, S. Shao, *Anal. Chem.* **2015**, *88*, 1455.

[32] C. Gao, Y. Tian, R. Zhang, J. Jing, X. Zhang, *Anal. Chem.* **2017**, *89*, 12945.

[33] H. Guo, G. Chen, M. Gao, R. Wang, Y. Liu, F. Yu, *Anal. Chem.* **2018**, *91*, 1203.
